# Multi-Dimensional Composite Frame as Bifunctional Catalytic Medium for Ultra-Fast Charging Lithium–Sulfur Battery

**DOI:** 10.1007/s40820-022-00941-2

**Published:** 2022-10-06

**Authors:** Shuhao Tian, Qi Zeng, Guo Liu, Juanjuan Huang, Xiao Sun, Di Wang, Hongcen Yang, Zhe Liu, Xichao Mo, Zhixia Wang, Kun Tao, Shanglong Peng

**Affiliations:** 1 National and Local Joint Engineering Laboratory for Optical Conversion Materials and Technology, School of Materials and Energy, Lanzhou University, Lanzhou, 730000 People’s Republic of China; 2 School of Physical Science and Technology, Lanzhou University, Lanzhou, 730000 People’s Republic of China

**Keywords:** MXenes, Transition metal sulfides, Lithium-ion transference, Bifunctional catalysis, Reaction kinetics

## Abstract

The shuttle effect of soluble lithium polysulfides (LiPSs) between electrodes and slow reaction kinetics lead to extreme inefficiency and poor high current cycling stability, which limits the commercial application of Li–S batteries. Herein, the multi-dimensional composite frame has been proposed as the modified separator (MCCoS/PP) of Li–S battery, which is composed of CoS_2_ nanoparticles on alkali-treated MXene nanosheets and carbon nanotubes. Both experiments and theoretical calculations show that bifunctional catalytic activity can be achieved on the MCCoS/PP separator. It can not only promote the liquid–solid conversion in the reduction process, but also accelerate the decomposition of insoluble Li_2_S in the oxidation process. In addition, LiPSs shuttle effect has been inhibited without a decrease in lithium-ion transference numbers. Simultaneously, the MCCoS/PP separator with good LiPSs adsorption capability arouses redistribution and fixing of active substances, which is also beneficial to the rate performance and cycling stability. The Li–S batteries with the MCCoS/PP separator have a specific capacity of 368.6 mAh g^−1^ at 20C, and the capacity decay per cycle is only 0.033% in 1000 cycles at 7C. Also, high area capacity (6.34 mAh cm^−2^) with a high sulfur loading (7.7 mg cm^−2^) and a low electrolyte/sulfur ratio (7.5 μL mg^−1^) is achieved.

**Supplementary Information:**

The online version contains supplementary material available at 10.1007/s40820-022-00941-2.

## Highlights

- The presence of Ti–O-Co bonds of the multi-dimensional composite frame separator (MCCoS/PP) promotes kinetics and enables bifunctional catalysis.
- The existence of MCCoS/PP cannot reduce the lithium-ion transference numbers.
- The Li–S battery with MCCoS/PP achieves super-high rate (368.6 mAh g^−1^ at 20C) and ultra-low capacity attenuation rate.

**Supplementary Information:**

The online version contains supplementary material available at 10.1007/s40820-022-00941-2.

## Graphical abstract

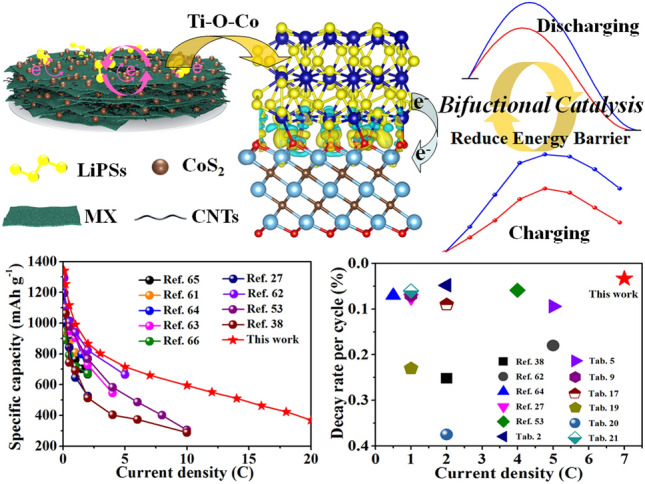

## Introduction

With the rapid development of portable electronic equipment, electric vehicles and large-scale energy storage, people need more diversified energy storage devices to replace traditional lithium-ion battery. Lithium–sulfur battery (Li–S battery) is considered as one of the best because of its high specific capacity (1672 mAh g^−1^), high energy density (2600 Wh kg^−1^), environmental characteristics and low cost [1]. However, Li–S battery is also facing severe problems, including poor rate performance and short cycle life. The root of these problems lies in the insulation of sulfur, the huge potential barrier needed for Li_2_S dissolution and the shuttle effect caused by soluble lithium polysulfides (LiPSs) [2–5]. Serious shuttle effect makes soluble LiPSs diffuse to lithium anode and deposit on lithium metal surface, which leads to the loss of active material sulfur. In addition, the slow sulfur redox reaction is also the key problem that limits the performance of Li–S battery [6–9].

In order to solve the above problems and improve the performance of Li–S battery, researchers have made great efforts. For example, Wu et al. reported that nitrogen-doped carbon was used as the substrate of sulfur cathode, and its large specific surface area was used to adsorb sulfur and LiPSs [10–13]. At the same time, the overall conductivity of composite was improved and the kinetics of sulfur redox reaction was accelerated to improve the utilization rate of cathode materials. In addition to the optimization of sulfur cathode, separator modification provides another simple method to achieve the above goals [14]. The active substance is filtered on the separator as a blocking layer to absorb the diffused LiPSs into the electrolyte. The LiPSs will realize the conversion of redox reaction on the separator material. This process not only causes the redistribution of active substance sulfur, but also gives full play to the adsorption and catalytic properties of separator modified material. Although the redox reaction kinetics of sulfur and LiPSs is greatly enhanced, the separator modification dramatically increases the transference path of lithium ions, which has a negative impact on the rate performance of Li–S battery. Therefore, finding suitable separator modification material and building a separator modification framework have become important topics to improve the performance of Li–S battery [15–19].

Recently, transition metal sulfide as separator modification material of Li–S battery has proved to have strong adsorption and catalytic ability for LiPSs [20–24], such as TiS_2_ [25], VS_2_ [26], CoS_2_ [27], and NiCo_2_S_4_ [28]. Among them, CoS_2_ is widely concerned because of its high binding energy with LiPSs, environmental characteristics and commercial feasibility. For example, Chen et al. reported that modified separator (AB-CoS_2_) with acetylene black and CoS_2_ composite showed good adsorption and catalytic activity when was applied to Li–S battery [29–32]. The Li–S battery with AB-CoS_2_ separator shows excellent rate performance (475 mAh g^−1^ at current density of 4C) and good cycle stability. However, the aggregation and structural collapse of transition metal sulfide catalysts often occur during charge and discharge, resulting in poor catalytic performance. An effective strategy to solve this problem is to assemble transition metal sulfides on conductive two-dimensional network to form composite structure, which can improve the stability of transition metal sulfides and enhance their adsorption and catalytic capabilities. MXene (MX) is a new two-dimensional transition metal carbon/nitride with the advantage of good conductivity, abundant surface functional groups and numerous active sites, which makes it one of the ideal separator modification materials for Li–S battery [18, 33–36]. Yang et al. proposed Ti_3_C_2_T_x_ composite GO as separator modification material and studied the ability of two different two-dimensional materials composite to block, capture and catalyze LiPSs [37]. However, when the two-dimensional (2D) sheet material is used as the modified material of Li–S battery separator, it is easy to stack between layers, which makes the transference path of lithium ion increase rapidly and the sluggish transfer of lithium ion at high rate [38]. In this work, carbon nanotubes (CNTs) were introduced during the preparation of the separator, and CNTs will exist between MX nanosheets, effectively keeping MX nanosheets from being re-stacked and providing guarantee with the rapid passage of lithium ions. To sum up, the growth of transition metal sulfides on the surface of MX nanosheets and the suction filtration with CNTs have been used to construct the multi-dimensional composite frame as the modificated separator for Li–S battery. The multi-dimensional composite frame separator can effectively inhibit the shuttle effect and accelerate the redox kinetics process, which shows great significance to boost the rate performance and cycle ability of Li–S battery [39–42].

In this paper, CoS_2_ nanoparticles have been refluxed on the alkali-treated 2D MX nanosheets to construct composite structure, and CNTs have been mixed for constructing a multi-dimensional composite frame as the modificated separator for Li–S battery. MX nanosheets as the main modification material of separator can not only effectively block LiPSs diffusion, but also prevent CoS_2_ nanoparticles from gathering and collapsing in the process of LiPSs conversion. The alkali-treated MX nanosheets have more oxygen-containing groups, which effectively promotes the growth of CoS_2_ on MX surface. However, since CoS_2_ nanoparticles are small in size, they cannot be used as scaffold to prevent MX nanosheets from stacking. CNTs have been introduced to support these layers, thus retain the active region needed for LiPSs catalysis and providing a channel for the rapid transference of lithium ions. Therefore, the multi-dimensional composite frame separator can not only promote the rate-controlling step of LiPSs conversion in the reduction process of Li–S battery, but also reduce the decomposition barrier of Li_2_S in the oxidation process. With a bifunctional catalytic activity, the multi-dimensional composite frame separator (MCCoS/PP) consisting of CoS_2_ nanoparticles on alkali-treated MX nanosheets and CNTs, provides important guidance on the next generation of Li–S battery with excellent efficiency and long cycling life.

## Experimental Section

### Preparation of MX@CoS_2_

#### ***Preparation of Ti***_***3***_***C***_***2***_***T***_***x***_*** Nanosheets***

Ti_3_AlC_2_ powders were bought from Beijing Forman Technology Co., Ltd. 0.8 g LiF powders (Aladdin Ar.) were dissolved in 9 M HCl and stirred for 20 min. 1 g Ti_3_AlC_2_ powders were slowly added to the above solution and stirred for 30 min. Heat the mixed solution to 35 °C and keep it for 48 h. Then centrifugally wash with deionized water at 3500 r until the pH value reached 6. The collection was ultrasonicated for 75 min under argon bubbling and centrifuged at 3500 r for 1 h. Collect supernatant and freeze drying.

#### ***Alkalization Treatment of Ti***_***3***_***C***_***2***_***T***_***x***_*** Nanosheets***

Dissolve a certain amount of Ti_3_C_2_T_x_ nanosheets in deionized water for ultrasonic treatment for 30 min, and add 1 M sodium hydroxide solution with the same volume to stand for 10 min. Samples were centrifugally washed with deionized water and freeze-dried.

#### Synthesis of Co Hydroxide Precursors

60 mg Ti_3_C_2_T_x_ nanosheets were added to the mixed solution of 4 mL deionized water and 12 mL ethylene glycol. Then, 150 mg Co(NO_3_)_2_·6H_2_O was added and ultrasonicated for 30 min. The solution was refluxed at 90 °C for 4 h in a flask under flowing N_2._ The product was washed with alcohol and deionized water three times and freeze-dried.

#### Synthesis of Vulcanization

The precursor powder was added to 0.5 M Na_2_S·9H_2_O (50 mL) and stirred for 30 min. The sample was transferred to a hydrothermal kettle and hydrothermal at 120 °C for 8 h. The product was washed with deionized water and freeze-dried.

### Synthesis of Separator

10 mg Ti_3_C_2_T_x_ was added with 20 mL isopropanol for ultrasonic treatment for 30 min and then added with 0.2 mL binder solution (PVDF is dissolved in NMP, mass fraction 2.5%) for ultrasonic treatment for 30 min. The mixed solution was filtered on Celgard 2400 by a vacuum filtration device and dried in vacuum at 60 °C for 8 h. The dried samples were cut into separator with a diameter of 19 mm. M/PP, MC/PP, and MCCoS/PP are prepared by the same method. (Samples with CNTs were prepared by the mass fraction of active substances and CNTs of 7:3.)

### Fabrication of the S Cathode

Typically, S powder and Ketjenblack (8:2 in weight) were mixed and sealed in a Teflon container filled with argon, followed by heating to 155 °C in an oven for 12 h. S/KB and water-based binder (LA132) are mixed and pestle according to the mass ratio of 9:1, coated on aluminum foil, and dried in vacuum at 60 °C for 12 h. The electrode is cut into 12-mm wafers, and the sulfur load of each wafer is 1.2–1.5 mg cm^−2^. The electrode with large load is the 1 × 1 cm^2^ carbon fiber cloth coated with active material. The calculation of the specific capacity is based on the mass of the active substance sulfur.

## Results and Discussion

### Synthesis of MX@CoS_2_ and Modified Separator

**Scheme 1:**
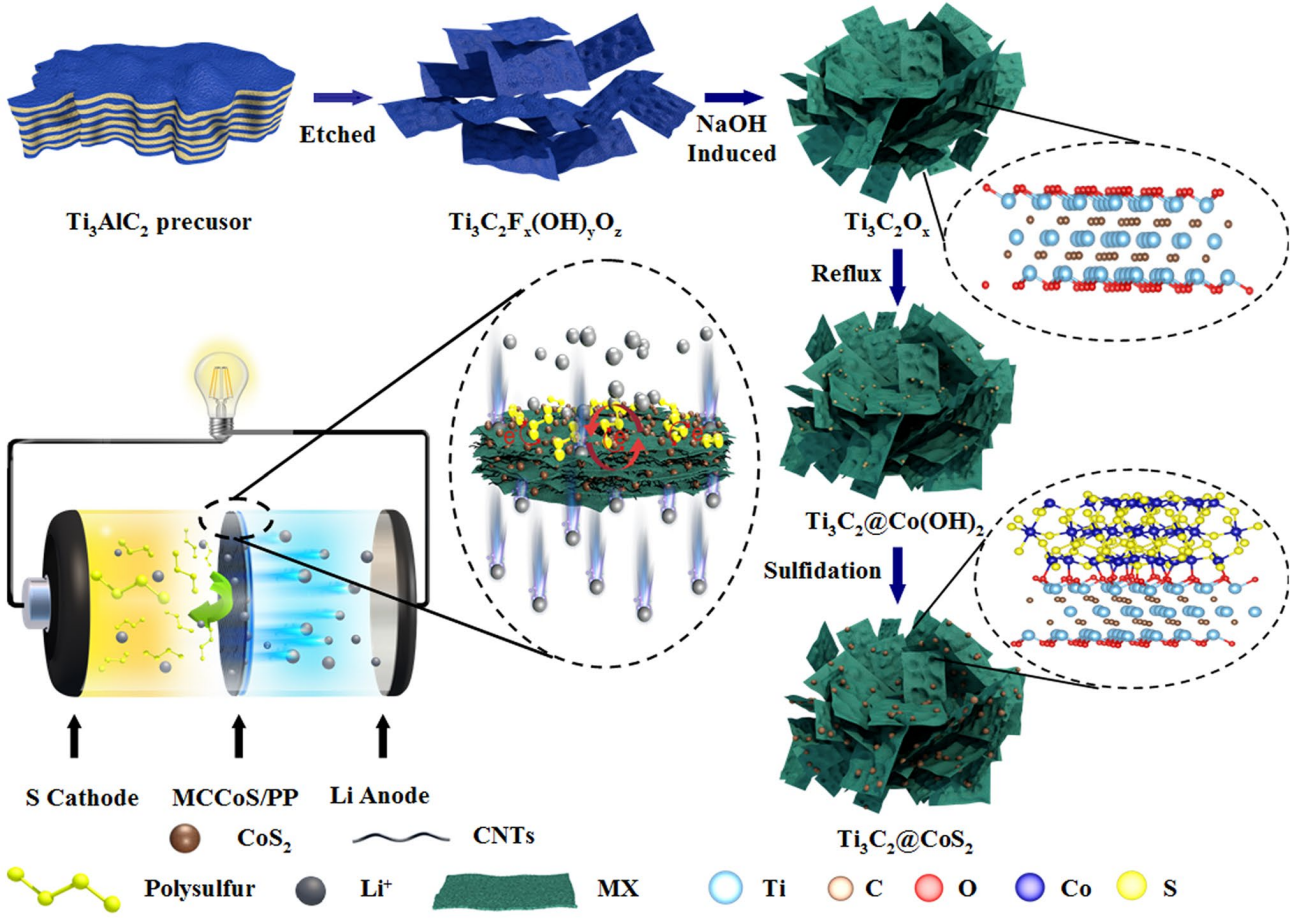
Synthesis process illustration of MX@CoS_2_ and Li–S battery configuration applying the multi-dimensional composite frame separator (MCCoS/PP)

Scheme 1 shows the synthetic route of MX@CoS_2_ and the architecture of the multi-dimensional composite frame separator. The accordion-shaped layered MX was prepared by the aluminum layer that was selectively etching in Ti_3_AlC_2_ precursor (Fig. S1a), and the surface of MX nanosheets after peeling was flat and smooth (Fig. S1b-c). The positively charged cobalt ions are uniformly adsorbed on the surface of MX nanosheets via electrostatic interaction, in which there are many oxygen-containing functional groups with electronegativity after alkali induction. Then, Co(OH)_2_ nanoparticles are uniformly grown on the surface of MX nanosheets under refluxing inert gas (Fig. S1d–e). After hydrothermal vulcanization, MX@CoS_2_ composite was successfully prepared (Fig. S1f). The prepared MX@CoS_2_ composite keeps the original sheet structure of MX nanosheets, and the composite structure with adjustable electronic characteristics was formed between CoS_2_ particles and MX nanosheets. Then, MX@CoS_2_ composite and CNTs were mixed and filtered on polypropylene separator (PP) to form a multi-dimensional composite self-supporting film as the modified layer of Li–S battery separator.

### Structure Characterization of MX@CoS_2_

**Fig. 1:**
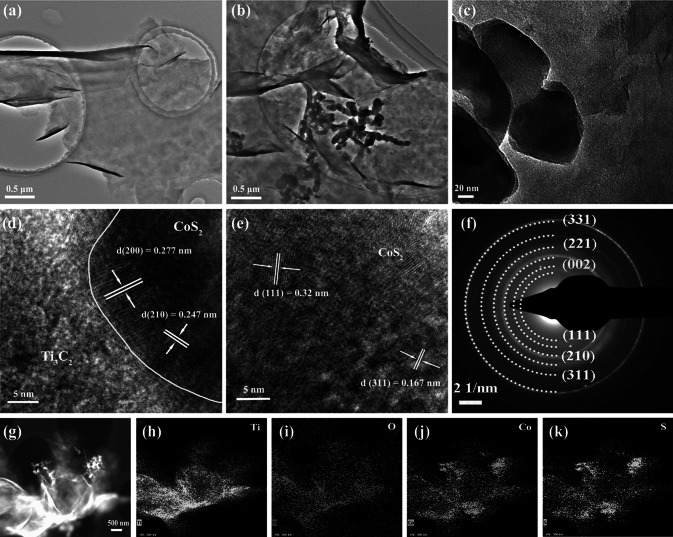
**a** TEM image of MX. **b**, **c** TEM image, **d**, **e** HRTEM image, **f** SAED patterns, and **g**–**k** HAADF-STEM image and the corresponding EDS elemental mapping of MX@CoS_2_

The TEM images of the morphology and microstructure of MX and MX@CoS_2_ composite are shown in Fig. 1a–c. Compared with the pure MX nanosheets, CoS_2_ nanoparticles with an average size of 50–80 nm are uniformly distributed on the surface of MX@CoS_2_ (Fig. S2). Lattice spacing of 0.277, 0.247, 0.32, and 0.167 nm can be observed from high-resolution TEM (HRTEM) (Fig. 1d–e), which corresponds to the (200), (210), (111), and (311) crystal planes of CoS_2_ nanoparticles [43]. Selected area electron diffraction (Fig. 1f) shows the concentric diffraction rings of CoS_2_ and MX, which can correspond to the crystal planes observed by HRTEM. Besides, the (002) crystal plane belonging to MX nanosheets is also found, which indicates the successful recombination of CoS_2_ and MX [44]. Elemental mapping analysis of high-angle annular dark field scanning transmission electron microscopy (HAADF-STEM) (Fig. 1g–k) shows that Ti, O, Co, and S are uniformly distributed on the surface of MX@CoS_2_ nanosheets, which further confirms the coexistence of CoS_2_ and MX.

**Fig. 2:**
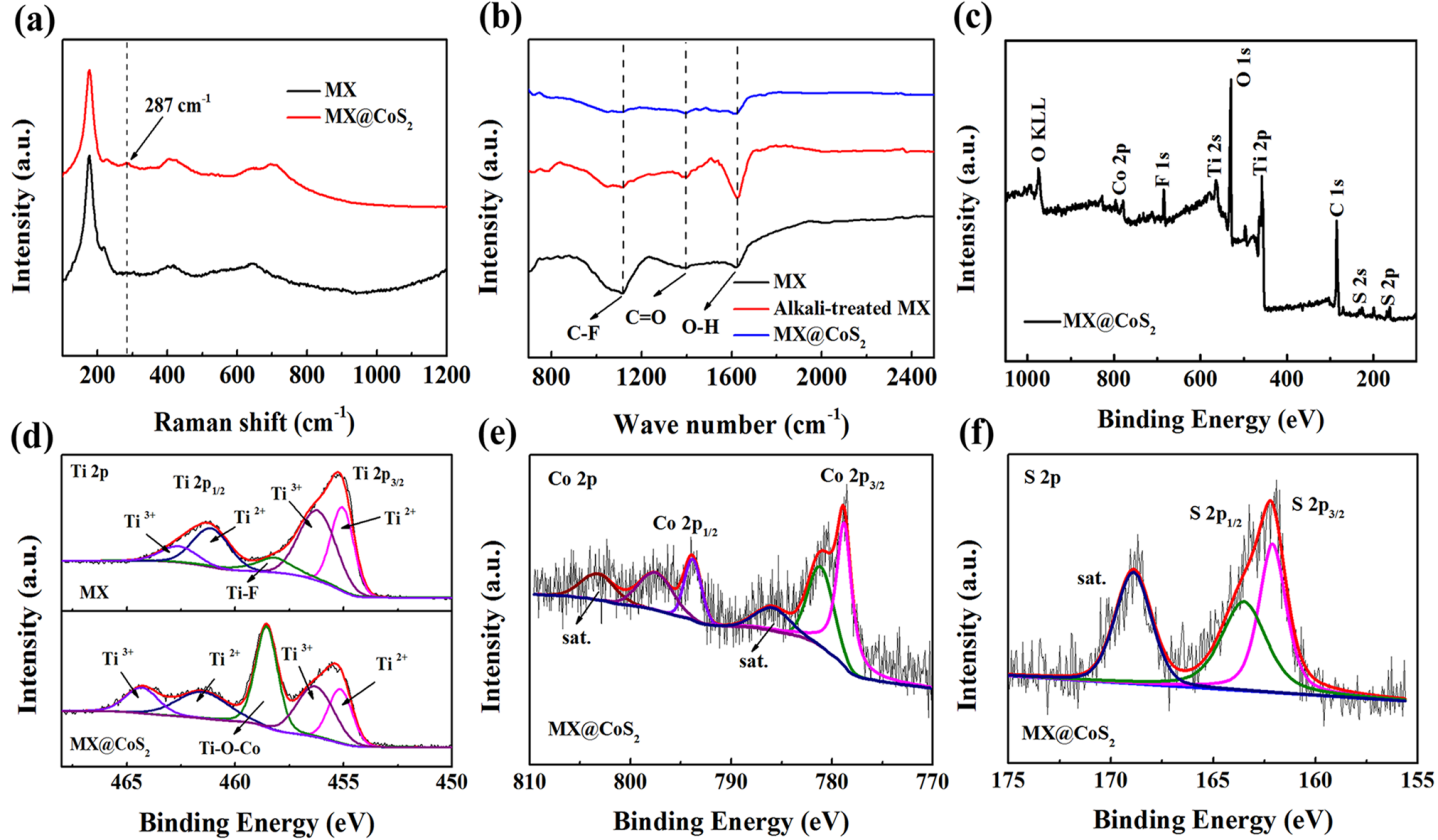
**a** Raman spectra, **b** FT-IR spectra of MX and MX@CoS_2_. **c** XPS survey spectra of MX@CoS_2_. **d** XPS Ti 2*p* spectra of MX and MX@CoS_2_. **e** XPS Co 2*p* and **f **XPS S 2*p* spectra of MX@CoS_2_

Further characterizations have been carried out to reveal the composite structure of MX@CoS_2_. The Raman spectrum of the MX nanosheets (Fig. 2a) exhibits peaks at 180, 220, 400, and 635 cm^−1^, which are attributed to titanium carbide MX [45]. In contrast, peaks from CoS_2_ (at 287 cm^−1^) were detected in the Raman spectrum of the MX@CoS_2_ [29]. The Fourier transform infrared (FTIR) spectra of MX nanosheets and the alkali-induced MX are given in Fig. 2b. It can be seen that after the alkali-induced treatment, the intensity of C-F (1115 cm^−1^) groups decreases significantly and the intensity of O–H (1397 cm^−1^) and C=O (1626 cm^−1^) groups is enhanced significantly [44]. This shows that alkali treatment of MX nanosheets can effectively increase oxygen-containing functional groups on MX surface. In addition, the peak value of each functional groups of MX@CoS_2_ composite decreases, which is caused by the growth of CoS_2_ on MX surface [44]. X-ray photoelectron spectroscopy (XPS) has been applied to observe the chemical state and the bonding of various elements in the MX@CoS_2_ composite. All the elements in MX and CoS_2_ can be seen from the whole spectrum (Fig. 2c), indicating the two-phase composite. The high-resolution spectrum of Ti 2*p* obtained from MX@CoS_2_ and MX is shown in Fig. 2d. The fitting peaks at 455.1 and 461.2 eV correspond to Ti^2+^, and the fitting peaks at 456.3 and 462.7 eV correspond to Ti^3+^ [46]. After alkali treatment, the fluorine functional groups on the surface of MX nanosheets are replaced by oxygen-containing functional groups. Ti–O–Co bonds are formed after the growth of CoS_2_ on alkali-treated MX, which can be verified by the fitting peak at 458.0 eV in Fig. 2d [47]. At the same time, the peak area ratio of Ti^2+^ and Ti^3+^ increases obviously, which is due to the electron transfer from CoS_2_ to MX and the formation of Ti–O–Co bond, which is consistent with the results of density functional theory (DFT) calculation below (in Fig. 4d). In Fig. 2e–f, the fitting peaks at 781.4 and 797.7 eV of Co 2*p* spectra correspond to Co–O bonds [48], which also confirms the formation of Co–O–Ti bonds. The fitting peaks at 778.9 and 161.8 eV of Co 2*p* and S 2*p* spectra can be attributed to Co-S and S-Co bonds in CoS_2_ [46, 49]. The above results indicate that MX and CoS_2_ are connected by oxygen-containing functional groups on MX surface and Ti–O–Co bonds are formed between the composite interfaces. Therefore, MX@CoS_2_ composite structure and electrons transfer are achieved.

### Ion Transference Characteristics and Catalytic Effects of MCCoS/PP

**Fig. 3:**
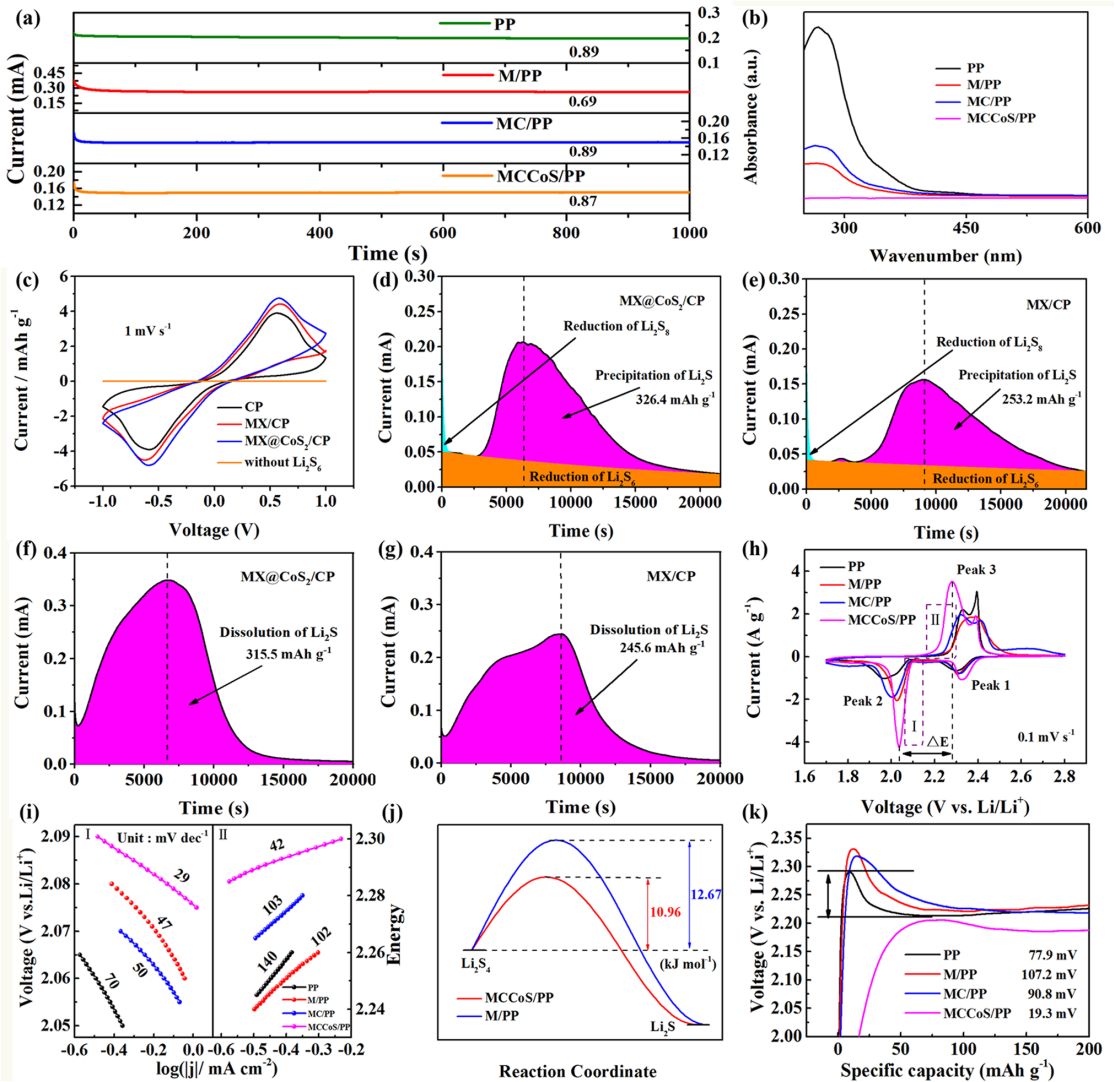
**a** Lithium-ion transference numbers for the PP, M/PP, MC/PP and MCCoS/PP separators tested by Li || Li symmetric cells. **b** UV–vis absorption spectra of the liquid in the right of electrolyzer. **c** CV curves of the symmetric cells assembled using CP, MX/CP, and MX@CoS_2_/CP. Precipitation profiles of Li_2_S with **d** MX@CoS_2_ and **e** MX. Dissolution profiles of Li_2_S with **f** MX@CoS_2_ and **g** MX. **h** CV curves. **i** Tafel plots calculated from the Peak 2 and Peak 3 of CV curves. **j** Activation energies (*E*a) of the Li_2_S_4_ reduction. **k** Charge curves of Li–S battery based on the different separators

MX@CoS_2_ composite as separator modification material is used to construct the separator for Li–S battery. Obstruction of lithium-ion transference and lengthy transference path are common problems in traditional Li–S battery separator modification materials [2]. Therefore, CNTs have been mixed with MX@CoS_2_ composite for constructing a multi-dimensional composite frame as the modificated separator for Li–S battery. CNTs exist between MX@CoS_2_ nanosheets, which plays a role in preventing nanosheets from stacking and reserving channels for the rapid passage of lithium ions. In addition, pure MX separator (M/PP) and MX mixed CNTs separator (MC/PP) have been also prepared as comparative samples. Then, the lithium-ion transference number test was carried out [2]. It can be seen from Fig. 3a that the transference number of PP separator is 0.89. Due to the stack of MX nanosheets (Fig. S3), MX separator has a value of 0.69. After CNTs have been introduced, the existence of CNTs between MX nanosheets provides a channel for the rapid passage of lithium ions (Fig. S4), so lithium-ion transference number of MC/PP is restored to 0.89. This scheme is also applied to MX@CoS_2_ composite, and its lithium-ion transference numbers is kept at 0.87. The rapid transference of lithium ions has laid a foundation for ultra-fast rechargeable Li–S battery.

The strong adsorption ability of LiPSs by separator modified material is the basic guarantee to suppress shuttle effect [29]. In order to evaluate the LiPSs adsorption capacity of MCCoS/PP separator, here, the H-type electrolyzer was designed and used to perform visual adsorption experiments of Li_2_S_6_ for different separator samples (Fig. S5). It can be seen that the common PP separator has no adsorption effect of Li_2_S_6_. After four hours, Li_2_S_6_ completely diffused from the left side to the right side of the electrolyzer, and the electrolyte changed color obviously. The MX nanosheets in the M/PP separator and MC/PP separator can promote the transformation of LiPSs into thiosulfates due to the existence of oxygen-containing functional groups on their surfaces, which has weak blocking ability for LiPSs. Therefore, the color of LiPSs on the right side become lighter at the same time. Since the CoS_2_ nanoparticles on the MCCoS/PP separator enhance the adsorption and catalysis of LiPSs on the separator, LiPSs do not diffuse to the right side of the electrolyzer even after 12 h. After 12 h, the liquid in the right of electrolyzer is sucked and tested by ultraviolet absorption spectrum (Fig. 3b). It can be seen that the absorption peak corresponding to LiPSs do not appear for the solution adsorbed by MCCoS/PP separator, which proves the strong adsorption capacity of MX@CoS_2_ for LiPSs [50]. The XPS (Fig. S6) of the adsorbed separator sample has been carried out, and it can be seen that the peak intensity ratio of Co^3+^ to Co^2+^ increases obviously, which indicates the electron transfer and strong adsorption capacity of MCCoS/PP separator for LiPSs [51].

After verifying the lithium-ion transference and LiPSs adsorption properties of the separator, the catalytic performance of the main material of the separator has been tested. Different separator samples were coated on carbon paper (CP) and assembled into symmetrical battery for testing (Fig. 3c). It can be seen that MX@CoS_2_/CP electrode shows higher current density and smaller polarization voltage than that of CP and MX/CP electrode, which indicates that MX@CoS_2_ composite structure can obviously promote the LiPSs conversion reaction [52]. The electrochemical impedance spectroscopy (EIS) of symmetrical battery (Fig. S7) shows that the charge transfer resistance of MX@CoS_2_/CP symmetrical battery is the smallest. It means that the kinetics of LiPSs conversion on the surface of MX@CoS_2_/CP has been effectively improved. The above tests show that MX@CoS_2_ composite can effectively adsorb LiPSs and further catalyze the transformation of LiPSs. In order to verify the influence of composite structure on the rate-controlling step of electrochemical reaction, the deposition experiment of Li_2_S has been further carried out (Fig. 3d–e). Compared with MX/CP electrode, the peak current of the Li_2_S deposition curve of MX@CoS_2_/CP electrode appears earlier, and the peak current is enhanced (0.21 mA at 6310 s for MX@CoS_2_ and 0.157 mA at 9074 s for MX), which indicates that the deposition speed of Li_2_S on the surface of MX@CoS_2_/CP electrode is faster. According to Faraday's law, the deposition capacity of Li_2_S on MX/CP and MX@CoS_2_/CP is 253.2 and 326.4 mAh g^−1^, respectively, which shows that MX@CoS_2_ can promote the deposition of Li_2_S. Therefore, Li_2_S deposition experiment proves that the conversion from Li_2_S_n_ to Li_2_S can be obviously enhanced, which is due to the catalytic effect of MX@CoS_2_. It is also important to evaluate the rate of Li_2_S dissolution on the catalytic matrix, which is another kinetic indicator for LiPSs conversion. As shown in Fig. 3f–g, the results show that MX@CoS_2_/CP exhibits earlier dissolution of Li_2_S and higher current density than MX/CP during the potentiostatic charge measurement (0.35 mA at 6634 s for MX@CoS_2_ and 0.246 mA at 8550 s for MX). In addition, the dissolving capacity of Li_2_S on MX@CoS_2_/CP (315.5 mAh g^−1^) is higher than that of MX/CP (245.6 mAh g^−1^). The above results show that MX@CoS_2_ can not only accelerate the rate-controlling step from Li_2_S_n_ to Li_2_S (reduction process), but also promote the dissolution of insoluble Li_2_S (oxidation process). This shows that the multi-dimensional composite frame separator has bifunctional catalytic effect on LiPSs conversion, which plays an important role in ultra-fast charge Li–S battery.

Electrochemical tests of Li–S battery have been further executed to evaluate the bifunctional catalytic effect of multi-dimensional composite frame separator (MCCoS/PP) on oxidation and reduction processes. In Fig. 3h, CV curves of Li–S battery modified by different separators are compared. Peak 1 and Peak 2 are attributed to the reduction of S_8_ to Li_2_S_4_ and the conversion of Li_2_S_4_ to solid Li_2_S, while Peak 3 represents the conversion of insoluble Li_2_S to S_8_. The peak intensities of the battery with MCCoS/PP separator are improved, and the potential difference between the oxidation peak and the reduction peak is reduced (Figs. 3h and S8), which indicates the reaction kinetics and reversibility of sulfur redox are facilitated. Tafel diagram based on CV curves (Fig. 3i) shows that the Li–S battery with MCCoS/PP separator has the smallest Tafel slope in both oxidation and reduction process, which also indicates the rapid transformation between LiPSs [53]. EIS (Fig. S9) further confirms this conclusion. The Li–S battery with MCCoS/PP separator has the smallest equivalent series resistance and charge transfer resistance. In addition, the activation energy from Li_2_S_4_ to Li_2_S (Fig. 3j) is calculated according to the Arrhenius formula:

1 $$j \propto A \times e^{{ - E_{a} /RT}}$$

where *j* refers to the peak current density, *A* refers to the pre-factor, *E*_*a*_ refers to the activation energy, *R* refers to the universal gas constant, and *T* refers to the Degree Kelvin [51]. By fitting the slope of the line at different temperatures, the calculated activation energy of the Li–S battery with MCCoS/PP separator is 1.71 kJ mol^−1^ lower than that of M/PP separator (Fig. S10). This reflects that the composite structure between CoS_2_ and MX promotes sulfur reaction kinetics in the reduction process. Figure 3k shows the influence of different separator samples on the decomposition energy barrier of Li_2_S from the charging curve. It can be seen that the charging curve "inverted V" of Li–S battery with MCCoS/PP separator is not obvious. This shows that MCCoS/PP separator plays a positive role in the decomposition of Li_2_S, which can greatly promote the oxidation process. Thus, the bifunctional catalytic effect of multi-dimensional composite frame separator on the redox process of Li–S battery is verified, and this shows great significance for ultra-fast charging Li–S battery.

**Fig. 4:**
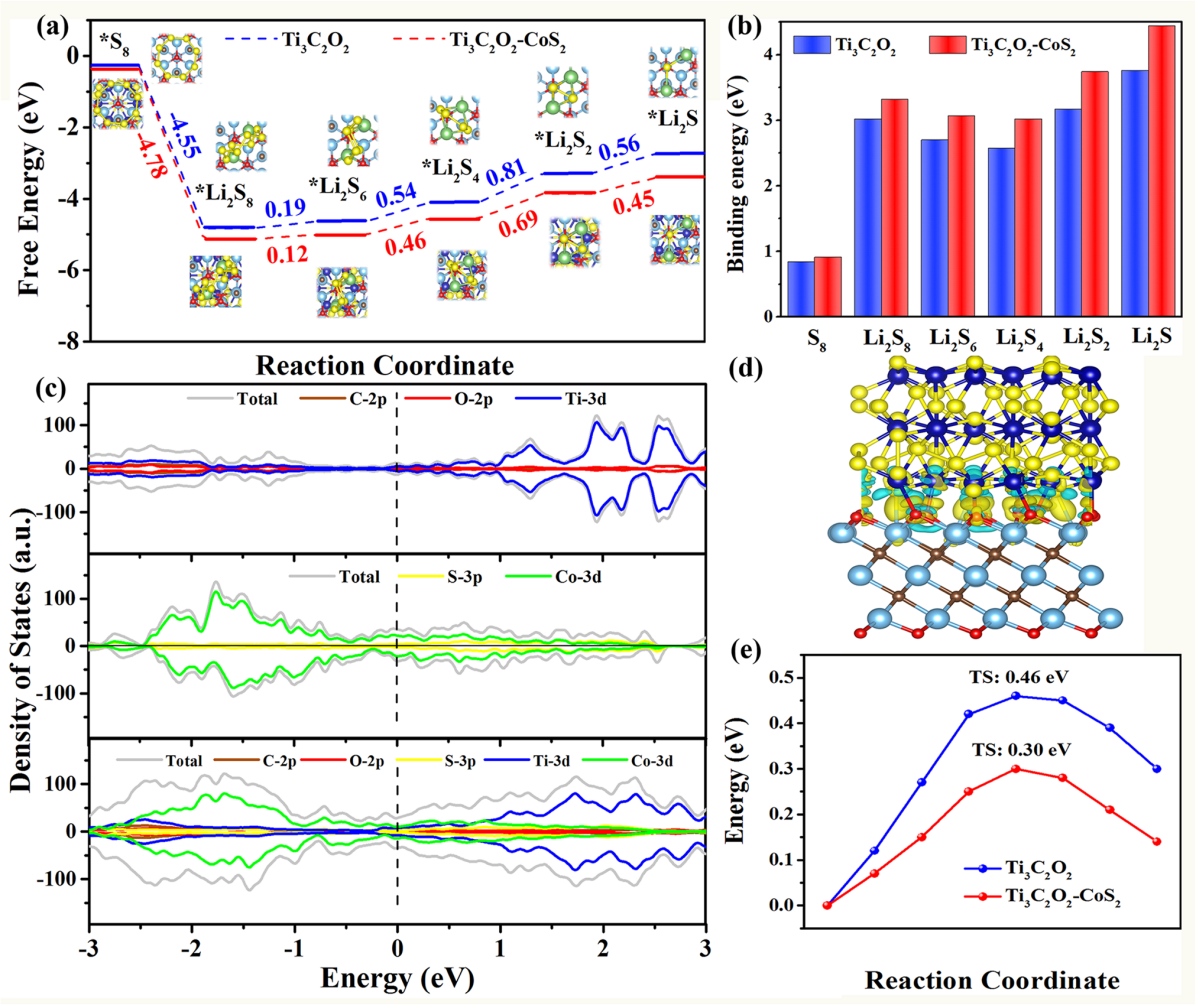
**a** The Gibbs free energy profiles of LiPSs on Ti_3_C_2_O_2_-CoS_2_ and Ti_3_C_2_O_2_. **b** Binding energies between LiPSs and Ti_3_C_2_O_2_-CoS_2_, Ti_3_C_2_O_2_ layers. **c** Calculated density of states of CoS_2_, Ti_3_C_2_O_2_ and Ti_3_C_2_O_2_-CoS_2_. **d** Differential charge density of Ti_3_C_2_O_2_-CoS_2_. **e** Energy profiles of Li_2_S decomposition on Ti_3_C_2_O_2_-CoS_2_ and Ti_3_C_2_O_2_

In order to further explain the electrocatalytic performance of MX@CoS_2_ composite at atomic level, DFT calculation has been carried out. Figure 4a shows the Gibbs free energy diagram of the reaction from S_8_ to Li_2_S. According to the calculation results of Gibbs free energy, it can be seen that the reaction from S_8_ to Li_2_S_8_ is spontaneous, and the transformation from Li_2_S_8_ to Li_2_S_6_ is close to thermodynamic equilibrium. Therefore, the slow redox reaction kinetics between sulfur and LiPSs in Li–S battery is mainly attributed to the transformation of Li_2_S_6_ to Li_2_S_2_ and Li_2_S. Under the catalysis of MX@CoS_2_, the reaction free energy of the rate-controlling step is only 0.46, 0.69, and 0.45 eV, which is far lower than that of the common MX (0.54, 0.84, and 0.56 eV) and the reported graphene (1.21 eV) [54]. This indicates that the presence of CoS_2_ on MX can effectively reduce the reaction barrier from Li_2_S_6_ to Li_2_S_2_ and Li_2_S [55]. In addition, the binding energy between MX@CoS_2_ and LiPSs is higher than that of MX (Fig. 4b), which means that the composite structure between CoS_2_ and MX can significantly enhance its adsorption of LiPSs [56]. This is consistent with previous electrochemical test results and visual adsorption experiment results.

Then, the density of states (DOS) of MX, CoS_2_, and MX@CoS_2_ has been calculated (Fig. 4c). It can be seen that the introduction of CoS_2_ increases the density of states at Fermi level and improves the overall conductivity of the composite, which is beneficial to charge transfer in the catalytic process [57, 58]. This can also be proved from the calculation results of differential charge density of MX@CoS_2_ composite (Fig. 4d). The calculation results show that CoS_2_ transfers electrons to MX, which reduces the valence state of Ti, resulting in the change of charge distribution center and the DOS. The increase in DOS at fermi surface can enhance the charge transfer between the composite and LiPSs, thus affecting the catalytic performance [50]. Figure 4e shows that the energy barrier of Li_2_S decomposition on MX@CoS_2_ (0.3 eV) is much lower than that on MX (0.46 eV), which is consistent with the experimental results of charge curve [52]. It shows that charge transfer at MX@CoS_2_ composite interface can effectively promote the decomposition of Li_2_S and accelerate the oxidation process of Li–S battery. To sum up, the introduction of CoS_2_ nanoparticles on common MX can not only enhance the interaction between MX@CoS_2_ and LiPSs, but also effectively promote the kinetics of LiPSs conversion reaction. This dynamic transition is bifunctional, which not only accelerates the rate-controlling step in the reduction process but also promotes the dissociation of Li_2_S in the oxidation process [59]. It shows that the MX@CoS_2_ composite structure is all-round to improve the performance of Li–S battery, especially for construction of ultra-fast charging Li–S battery.

### Electrochemical Performance of Li–S Batteries with MCCoS/PP

**Fig. 5:**
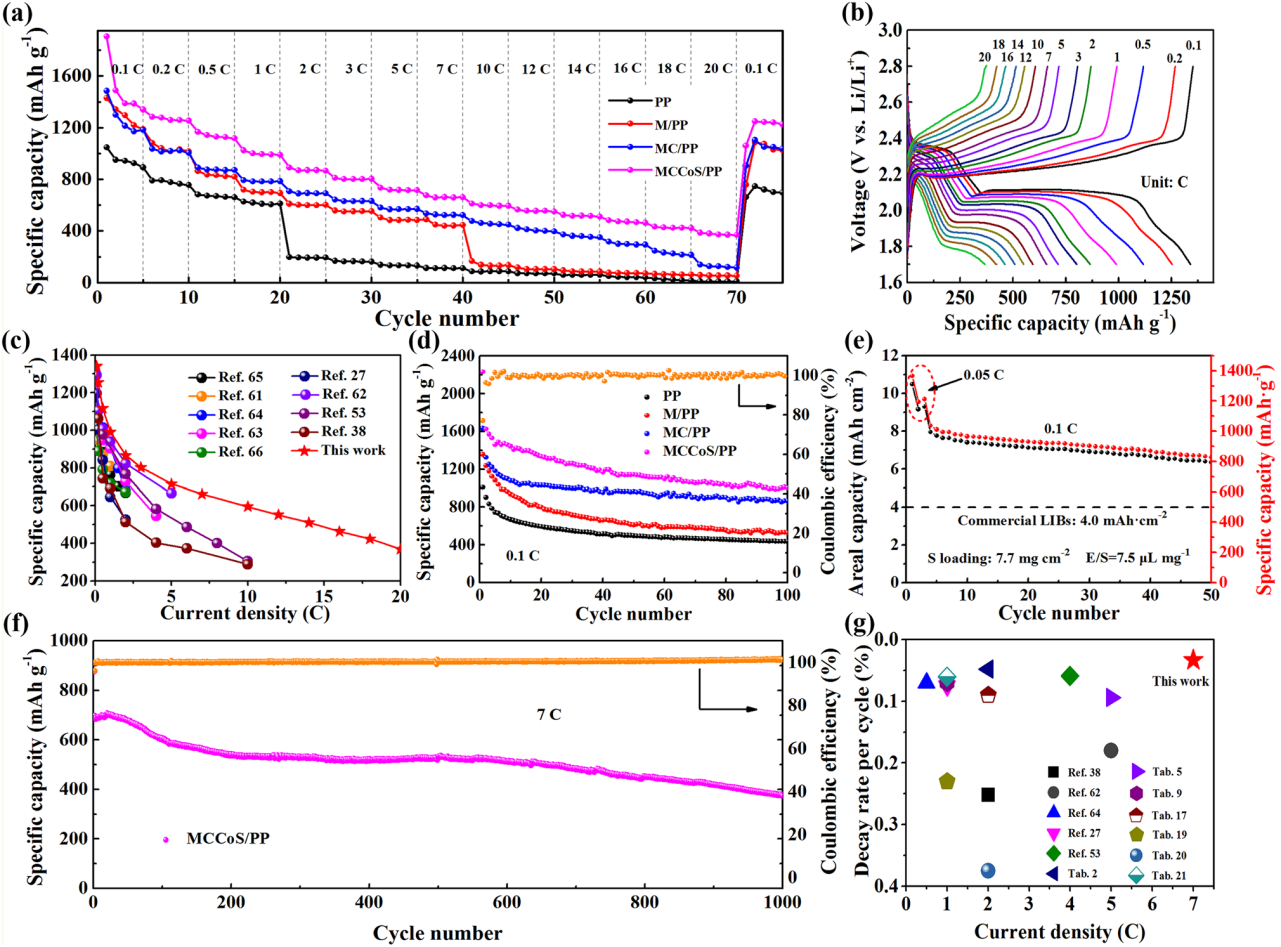
Electrochemical performances. **a** Rate performance of the different separators. **b** The galvanostatic charge–discharge profiles of Li–S battery based on MCCoS/PP separator at different current densities. **c** Comparison of MCCoS/PP as separator of Li–S battery with other MX based materials in rate performance. **d** Cycling performance at 0.1C of Li–S battery based on different separators and **e** cycling performance of Li–S battery based on MCCoS/PP separator at a high sulfur loading and low electrolyte/sulfur ratio. **f** Long-cycle performance at 7C of Li–S battery based on MCCoS/PP separator. **g** Comparison of MCCoS/PP as separator of Li–S battery with other works in long-cycle performance

Due to the bifunctional catalytic activity of MCCoS/PP separator, the redox transformation of LiPSs is promoted, and the ion diffusion in active materials (Figs. S11 and S12) is enhanced [60]. The Li–S battery with multi-dimensional composite frame separator exhibits super-high rate capability and ultra-fast charging. The rate performance of Li–S battery with various separators at different current densities from 0.1 to 20 C (1C = 1672 mAh g^−1^) has been tested (Fig. 5a). The battery with MCCoS/PP separator provides high specific capacity of 1340.7, 1117.2, 866.5, 715.3, 661.2, and 368.6 mAh g^−1^ at current densities of 0.1, 0.5, 2, 5, 10, and 20C, and when current densities return to 0.1C, it has a specific capacity of 1241.3 mAh g^−1^. In contrast, the rate performance of Li–S battery with other separators is not satisfactory. It can be seen that the battery with MCCoS/PP separator can maintain a good charge–discharge platform even at the ultra-high current density of 20C (Fig. 5b). Due to the capacity contribution of the separator material itself, the first few cycles of the battery equipped with the MCCoS/PP separator at a current density of 0.1C exceeded the theoretical specific capacity. To quantify the impact of the separator material on the test, lithium-ion batteries (LIBs) were assembled and operated within the same voltage window as Li–S batteries. The detailed information is described in Supporting information, and the result is shown in Figs. S13 and S14. After removing the extra capacity, the Li–S battery equipped with the MCCoS/PP separator still shows an ultrafast charging capability superior to the batteries equipped with other separators. Compared to the previously reported Li–S battery with other MX-based materials (Fig. 5c and Table S1), the Li–S battery with MCCoS/PP separator (this work) has higher specific capacity than other types of battery either at low current density or at high current density [27, 38, 53, 61–66]. Especially when the current density is greater than 10C, similar Li–S battery cannot bear it due to the slow kinetics and ion transference, but the battery with MCCoS/PP separator can still continue to work and provide a specific capacity of 368.6 mAh g^−1^ even at 20C. It is impossible to achieve excellent rate performance only by accelerating kinetics or realizing rapid ion transmission (Fig. S15). All these are attributed to the combined action of bifunctional catalysis and rapid ion transmission of multi-dimensional composite frame separator, thus achieving a great breakthrough in rate performance.

In addition to excellent rate performance, the multi-dimensional composite frame separator also promotes the reversibility and the cycling performance of Li–S battery. During the cycling processes, MCCoS/PP separator makes sulfur redistribute on the composite structure. Sulfur is dispersed and fixed on the composite structure separator, which inhibits the shuttle effect of LiPSs, improves the utilization of active materials and slows down the growth rate of lithium dendrites (Figs. S16 and S17), so that the cycle stability and coulombic efficiency (CE) (Fig. S18) of the battery with MCCoS/PP separator are also very prominent compared with similar Li–S battery. Figure 5d shows the cycling performance at 0.1C. It can be seen that the battery with MCCoS/PP separator has higher capacity than other separator samples, even after removing the capacity contribution of the separator material (Fig. S19). The cycling performance of the MCCoS/PP modified battery with high sulfur loading and low electrolyte/sulfur (E/S) has been also evaluated (Fig. 5e). The MCCoS/PP modified battery shows high specific capacity of 826.1 mAh g^−1^, high areal capacity of 6.34 mAh cm^−2^ at 0.1C and high stability even under high sulfur load (7.7 mg cm^−2^) and low electrolyte/sulfur ratio of 7.5 μL mg^−1^. Its area capacity is 1.5 times that of commercial lithium-ion battery (4 mAh cm^−2^), which shows great significance to the commercialization of Li–S battery. Finally, the long-cycle performance of Li–S battery with MCCoS/PP separator was tested at a high current density of 7C (Fig. 5f). It is found that the battery with MCCoS/PP separator shows a high initial discharge capacity of 698.1 mAh g^−1^, and after 1000 cycles, the capacity decay per cycle is only 0.033%. Moreover, the CE of the battery remains at 99%-100% for 1000 cycles. The excellent cycling performance not only comes from the excellent adsorption and catalysis of LiPSs by MX@CoS_2_ composites, but also benefits from the existence of CNTs. The existence of CNTs avoids the stacking of sheet-like structures and provides more LiPSs adsorption and catalytic sites, which are also important for the improvement of the energy density and cycle retention of Li–S battery. Compared with the similar work (Fig. 5g and Table S1), this work can achieve the lower capacity attenuation and long-cycle performance at higher current density.

## Conclusions

The multi-dimensional composite frame, composed of CoS_2_ nanoparticles on alkali-treated MX nanosheets and CNTs, has been proposed as the bifunctional catalytic modified separator of Li–S battery. Ti–O–Co bonds and electron transfers from CoS_2_ to MX are observed in the MX@CoS_2_ composite structure, which are also verified by the differential charge density calculation. The MX@CoS_2_ composite structure improves the adsorption and catalytic abilities of the separator to LiPSs and then effectively inhibits the LiPSs shuttle effect and accelerates the oxidation–reduction reaction kinetics of sulfur and LiPSs. CNTs in the multi-dimensional composite frame separator guarantee channels for the rapid passage of lithium ions by preventing nanosheets from stacking. Based on the experiments and theoretical calculations, it is found that the multi-dimensional composite frame separator can not only promote conversion from Li_2_S_n_ to Li_2_S in the reduction process, but also effectively reduce the decomposition barrier of Li_2_S in the oxidation process. This is attributed to the effect of the composite structure between MX and CoS_2_ on the overall density of states of the system. Due to the bifunctional catalysis and rapid ion transmission of multi-dimensional composite frame separator, the Li–S battery with MCCoS/PP separator achieves super-high rate performance with a specific capacity of 368.6 mAh g^−1^ at 20C. During the cycling processes, MCCoS/PP separator makes sulfur redistribute on the composite structure, and sulfur is dispersed and fixed on the composite structure separator. The Li–S battery also shows ultra-low capacity attenuation rate of 0.033% in 1000 cycles at 7C. This work may provide important guidance for the next generation of Li–S battery with high power density and long cycling life.

## Supplementary Information

Below is the link to the electronic supplementary material.

Supplementary file1 (PDF 2722 kb)
