# Supplementary material for: Multi-Dimensional Composite Frame as Bifunctional Catalytic Medium for Ultra-Fast Charging Lithium–Sulfur Battery
Source: Nanomicro Lett. 2022 Oct 6;14:196. doi: 10.1007/s40820-022-00941-2 (PMC9537413; doi:10.1007/s40820-022-00941-2)
Supplement: Supplementary file 1 — Supplementary file1 (PDF 2722 kb) [file 40820_2022_941_MOESM1_ESM.pdf]

Supporting Information for

Multi-Dimensional Composite Frame as Bifunctional Catalytic Medium for Ultra-Fast Charging Lithium-Sulfur Battery

Shuhao Tian[1, #], Qi Zeng[1, #], Guo Liu[2], Juanjuan Huang[1, *], Xiao Sun[1], Di Wang[1], Hongcen Yang[1], Zhe Liu[1], Xichao Mo[2], Zhixia Wang[1], Kun Tao[2], Shanglong Peng[1, *]

[1] National & Locai Joint Engineering Laboratory for Optical Conversion Materials and Technology, School of Materials and Energy, Lanzhou University, Lanzhou 730000, P. R. China

[2] School of Physical Science and Technology, Lanzhou University, Lanzhou 730000, P. R. China

[#] Shuhao Tian and Qi Zeng contributed equally to this work.

*Corresponding authors. E-mail: pengshl@lzu.edu.cn (Shanglong Peng), huangjj@lzu.edu.cn (Juanjuan Huang)

S1 Preparation of Related Solutions and Testing Method

Materials characterizations: The structures and morphologies of the prepared samples were characterized by field-emission scanning electron microscopy (FE-SEM, Apero S) and high-resolution transmission electron microscopy (HRTEM, FEI Tecnai G2 F30). The crystal structure of the samples was identified by X-ray powder diffraction (XRD, Rigaku RINT2400 with Cu Kα radiation). X-ray photoelectron spectroscopy (XPS) analysis was carried out using a Kratos Axis Ultra DLD instrument with Al Kα probe beam.

Preparation of the Li_2S_6 and Li_2S_8 solution: Li_2S_6 and Li_2S_8 were selected as prototype of LiPS. The 0.5 M Li_2S_6 solution was prepared by mixing sulfur and Li_2S (5:1 in weight) in 1,3-dioxolane (DOL) and dimethoxymethane (DME) (1:1, v/v) mixed solvent, followed by stirring at 60 °C for 24 h. 1 M Li_2S_8 is also configured according to the above method, but the solvent is changed to ethylene glycol dimethyl ether. In adsorption and cell test, the difference between LiPS is whether to add 1 M LITFSI or not. In addition, Li_2S_6 and Li_2S_8 blank electrolyte is also configured.

LiPS adsorption tests: Add 20 mL Li_2S_6 (3 mM) to one side of the electrolyzer and 20 mL blank electrolyte to the other side, and sandwich the modified diaphragm in the middle of the electrolyzer. After the adsorption test, absorb the electrolyte on the blank side for UV-vis spectroscopy, and the active material can be used for XPS analysis.

Assembly and tests of symmetric cells: Dissolve 5 mg of active substance in 10 mL of ethanol, ultrasonic for 30 min, measure the corresponding solution with a pipette and drop it on carbon paper to prepare electrode materials. Add 20 μL Li_2S_6 (0.5 M) to both sides of the diaphragm to assemble symmetrical batteries for electrochemical test, and the voltage window is -1 – 1 V. EIS measurements were conducted in the frequency range of 0.01 Hz to 10 kHz.

Li_2S precipitation measurement: The electrode material is prepared by the above method. The electrode material and lithium sheet are used to assemble the battery, and 20 μL blank electrolyte is dripped on one side of the lithium sheet and 10 μL Li_2S_8 (0.3 M) is dripped on one side of the electrode material. The lithium-catalyst batteries were galvanostatically discharged at 0.112 mA to 2.06 V, and then potentiostatically discharged at 2.05 V for 21,600 s.

Li_2S dissolution measurement: The electrode material is prepared by the above method. The electrode material and lithium sheet are used to assemble the battery, and 20 μL blank

electrolyte is dripped on one side of the lithium sheet and 10 μL Li_2S_8 (0.3 M) is dripped on one side of the electrode material. The lithium-catalyst batteries were galvanostatically discharged at 0.1 mA to 1.7 V, and then potentiostatically discharged at 2.35 V for 20000 s.

Lithium ion diffusion measurement: Cyclic voltammetry tests were carried out at different sweep speeds of 0.1 to 0.5 mV s^{-1} (Figure 4b, S8 and S9) to study the lithium ion diffusion caused by different samples on the electrode surface. There is a linear relationship between the current density of two reduction peaks and one oxidation peak in lithium-sulfur battery and the square root of scanning rate, which can be fitted by Randles-Sevcik equation:

$$I_p = (2.69\times10^5)\cdot n^{1.5}\cdot A\cdot D^{0.5}\cdot C_{Li+}\cdot v^{0.5} \quad \text{(S1)}$$

where I_P is the peak current density, n is the number of electrons in the reaction, A is the active electrode area, D is lithium ion diffusion coefficient, C_{Li+} is the lithium ion concentration and v is the scanning rate [54].

Cell assembly and measurements: CR2032-type button were assembled and sealed in a high-purity arogon-filled glovebox (H_2O, O_2 <0.01 ppm) Lithium foil (15.6 mm) was used as anode and S@C electrode as cathode. The electrolyte was prepared by dissolving 1 M LiTFSI and 2.0 wt% $LiNO_3$ in DME and 1,3-dioxolane (DOL) (1:1, v/v). The electrolyte amount added in the common cell is 40 μL. The galvanostatic charge-discharge test was carried on a LAND battery tester in voltage range between 1.7 - 2.8 V. CV and EIS measurements (0.01-10^5 KHz) were performed on the CHI-760E electrochemical workstation.

DFT calculations: All the spin-polarized density functional theory (DFT) calculations were performed by using the projected augmented wave (PAW) pseudopotentials, as implemented in the Vienna ab-initio Simulation Package (VASP). The generalized gradient approximation (GGA) in the Perdew-Burke-Ernzerhof (PBE) form was employed. The cut-off energy was set to 450 eV and the convergence threshold was set to 10^{-5} and 0.02 eV Å^{-1} for energy and force, respectively. The van der Waals (vdW) interactions between $Ti_3C_2O_2$ and Li_2S_n were dealt with using the Grimme's D3-type of the semiempirical method. A 4×4×1 k-mesh was employed for the Brillouin zone integrations, and a vacuum layer of 15 Å was employed to avoid interactions of neighbouring images. In addition, the climbing nudged elastic band method (CNEB) method was used for computing Li_2S decomposition barriers. To construct the surface slab models, (4×4) $Ti_3C_2O_2$ and the (111) surface of (√3×√3) CoS_2 supercell were used with the total 202 atoms.

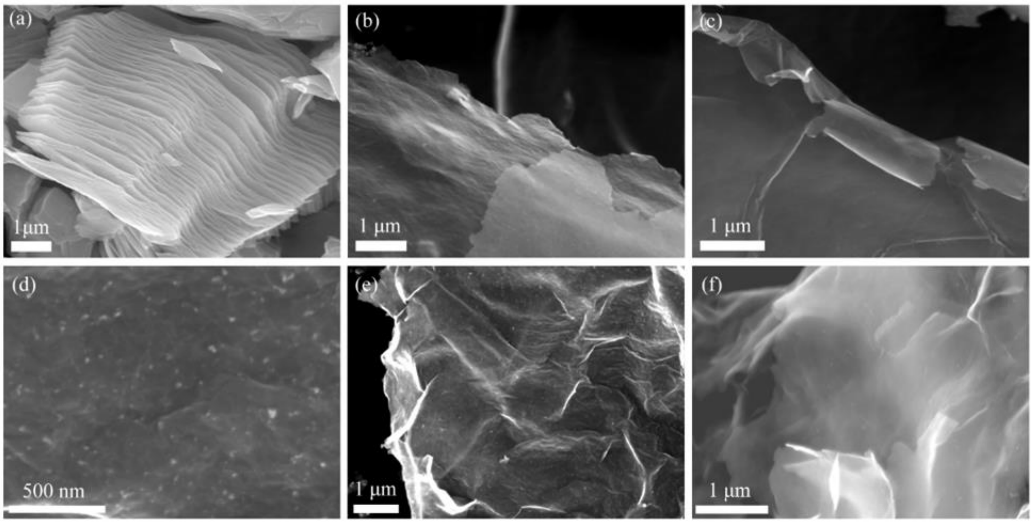

Fig. S1 a) SEM image of MX, **b-c**) SEM images of MX nanosheets, **d-e**) SEM images of MX@ $Co(OH)_2$, f) SEM image of MX@CoS_2

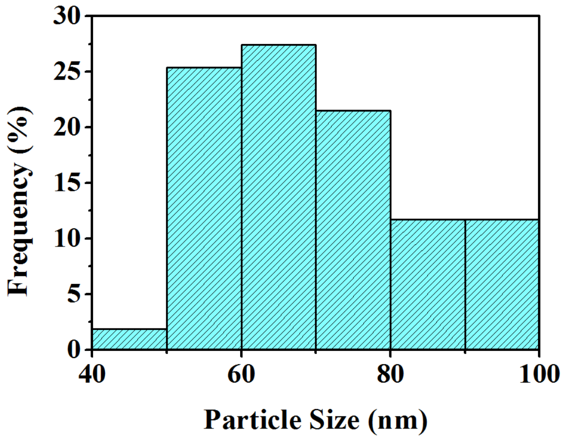

Fig. S2 The size distribution of CoS_2 nanoparticles obtained from Figure 1b

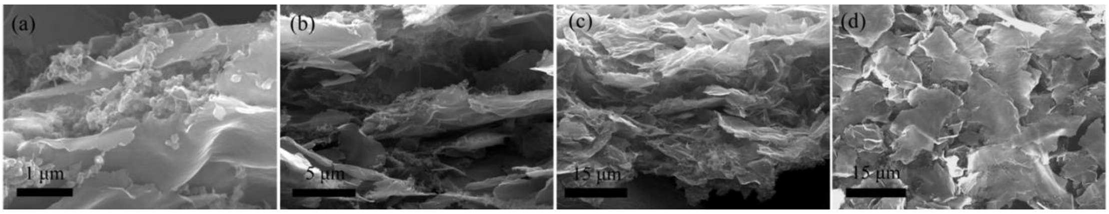

Fig. S3 a-c) A typical sectional view of the M/PP modified separator, **d**) top view

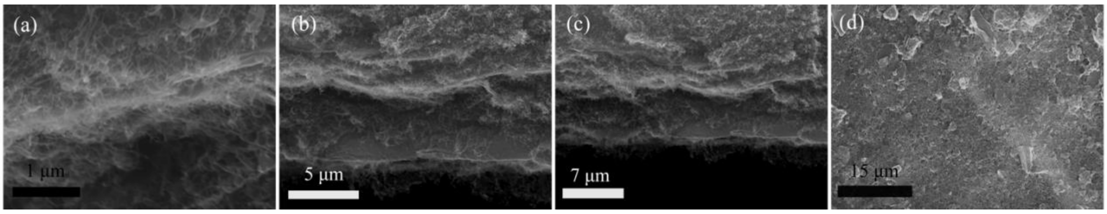

Fig. S4 a-c) A typical sectional view of the MCCoS/PP modified separator, **d**) top view

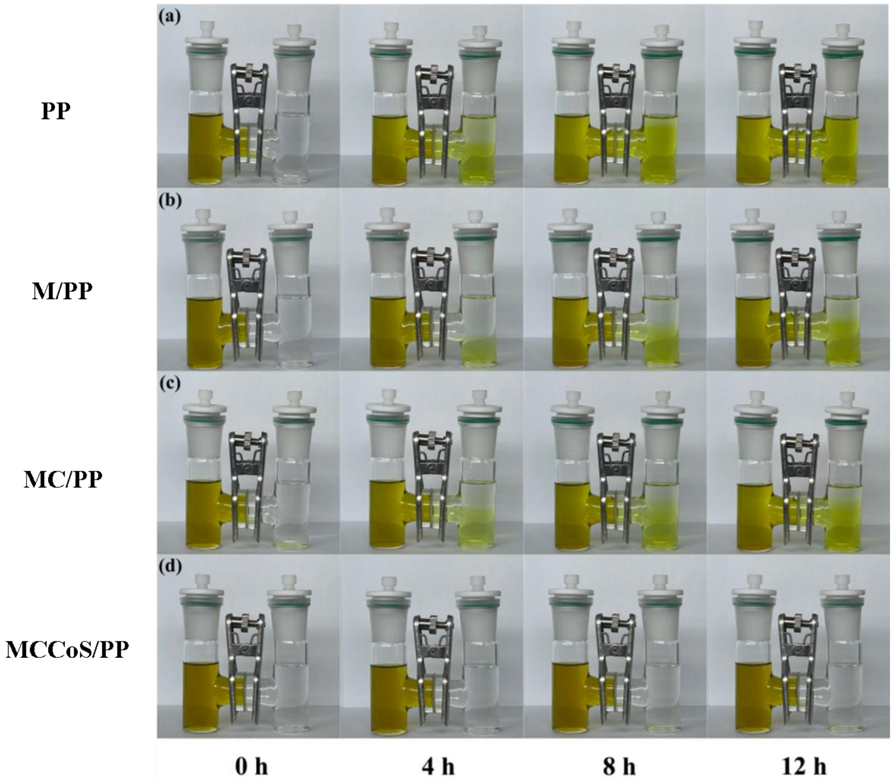

Fig. S5 Diffusion tests of Li_2S_6 with **a**) PP, **b**) M/PP, **c**) MC/PP, and **d**) MCCoS/PP separator

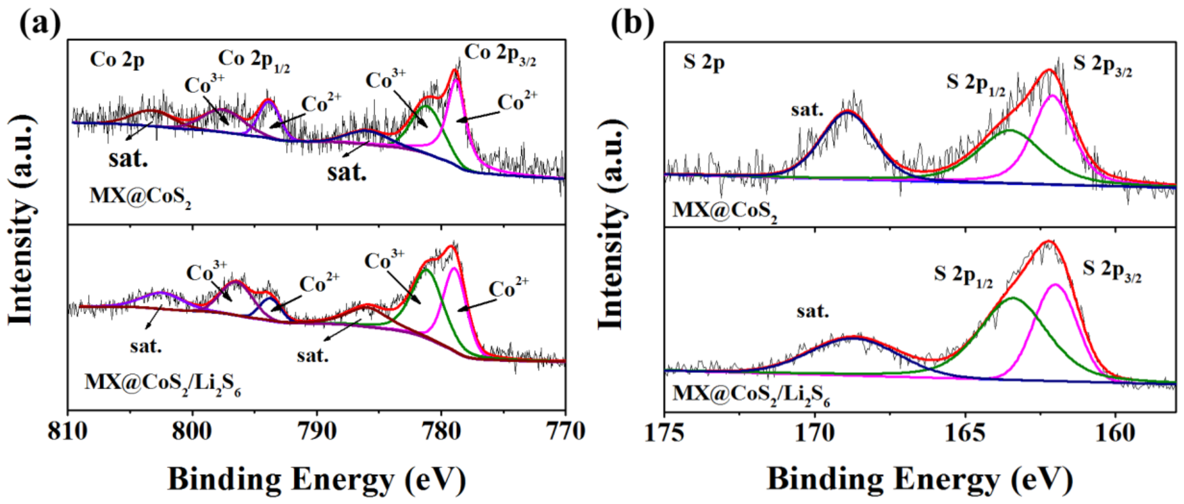

Fig. S6 a) XPS Co 2p and **b**) XPS S 2p spectra of MX@CoS_2 after Li_2S_6 adsorption

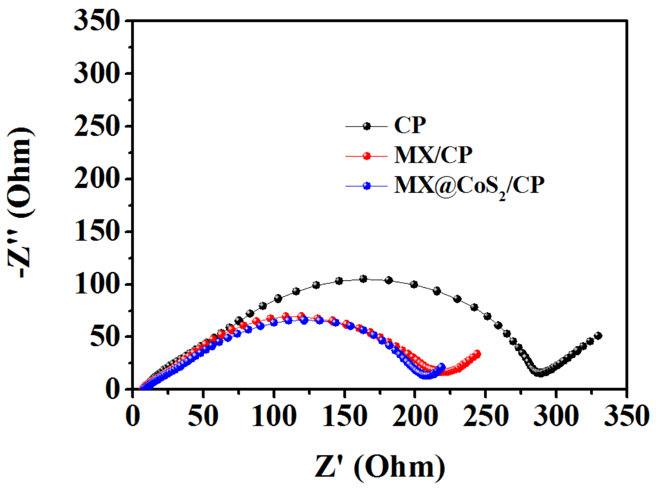

Fig. S7 EIS spectra of the symmetric cells assembled using CP, MX, and MX@CoS_2

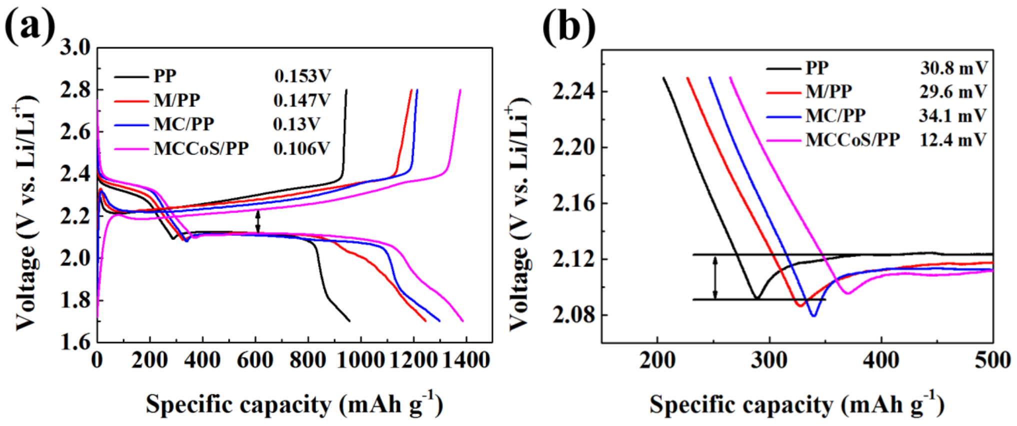

Fig. S8 a) XPS Co 2p and **b**) XPS S 2p spectra of MX@CoS_2 after Li_2S_6 adsorption

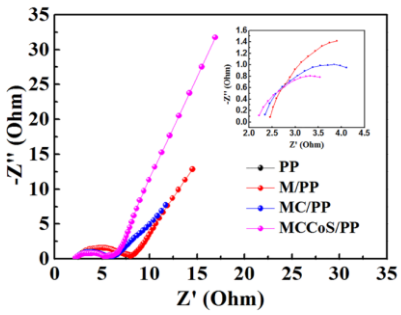

Fig. S9 EIS of Li–S battery based on the different separators

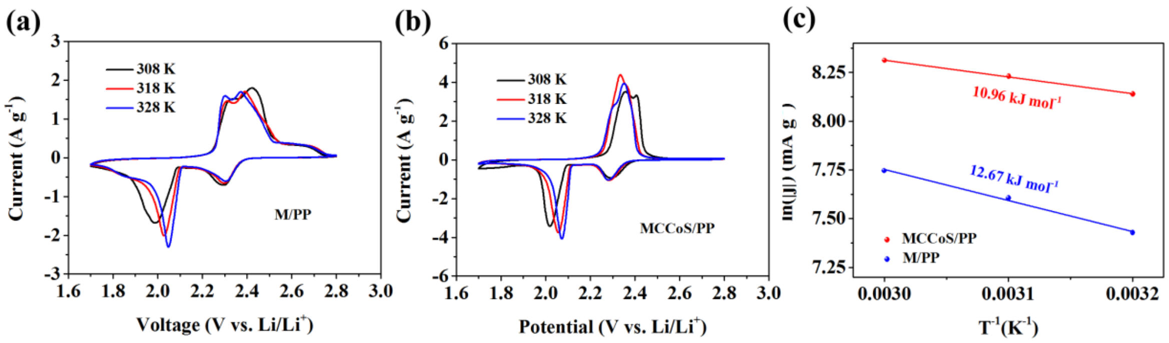

Fig. S10 CV curves of the Li–S batteries using (**a**) M/PP, and (**b**) MCCoS/PP separator at various temperatures. (**c**) Relation of Li_2S_4 conversion reaction with respect to temperatures of Li-S battery based on M/PP and MCCoS/PP separator

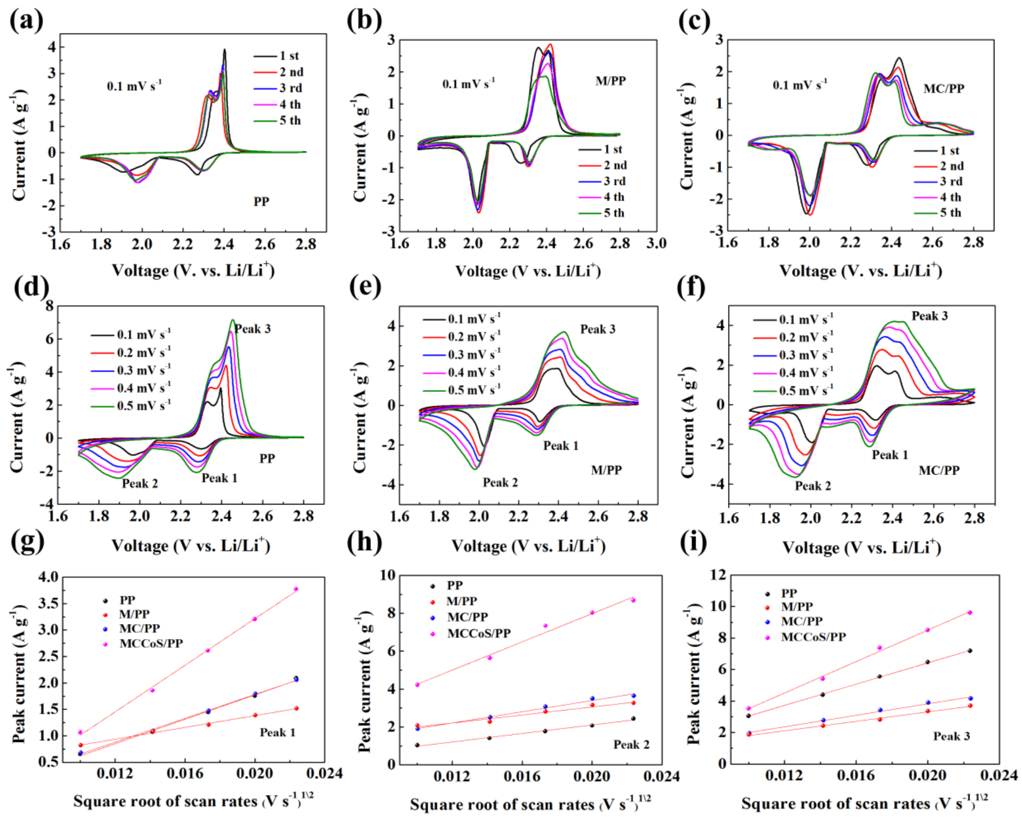

Fig. S11 a-c) CV curves of Li–S battery based on the different separators, **d-e**) CV curves of Li–S battery based on the different separators at various scan rates, **g-i**) Plot of CV peak of **g**) Peak 1 (S_8-Li_2S_4), **h**) Peak 2 (Li_2S_4-Li_2S), and i) Peak 3 (Li_2S-S_8) versus the square root of scan rates

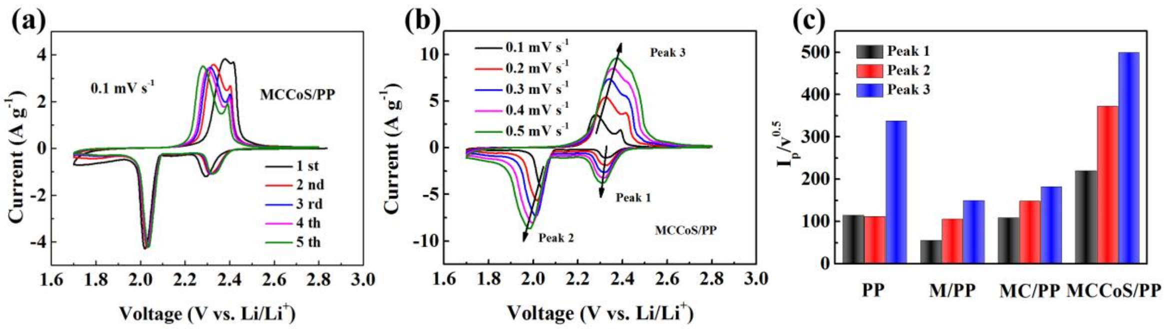

Fig. S12 a) CV curves of Li–S battery based on the MCCoS/PP separator **b**) CV curves of Li–S battery based on the MCCoS/PP separator at different scan rates from 0.1 to 0.5 mV s^{-1}. **c**) Values of CV peak current (I_p)/square root of the scan rates ($v^{0.5}$) for the four different separators in the first (peak A: $S_8 \rightarrow Li_2S_x$) and second (peak B: $Li_2S_x \rightarrow Li_2S_2/Li_2S$) cathodic reduction processes and the anodic oxidation process (peak C: $Li_2S_2/Li_2S \rightarrow S_8$)

S2 Assembling, Electrochemical Tests, and Analysis of LIBs

The Ti_3C_2@CoS_2/CNTs and PVDF were mixed at a mass ratio of 9:1 and ground for 1 h, and then NMP was added for grinding for 0.5 h. The obtained samples were coated on aluminum foil and dried under vacuum at 60 °C for 24 h. The obtained sample was cut into electrode disks with a diameter of 12 mm. CR2032-type button were assembled and sealed in a high-purity arogon-filled glovebox (H_2O, O_2 <0.01 ppm). Lithium foil (15.6 mm) was used as anode and Ti_3C_2@CoS_2/CNTs electrode as cathode. The electrolyte was prepared by dissolving 1 M LiTFSI and 2.0 wt% $LiNO_3$ in DME and 1,3-dioxolane (DOL) (1:1, v/v). The electrolyte amount added in the common cell is 40 µL. The galvanostatic charge-discharge test was carried on a LAND battery tester in voltage range between 1.7 - 2.8 V. The applied current values are based on Li-S batteries to guarantee the accuracy of the data. That is, in the cycling performance test, the current density of the Li-S battery is set to 0.1C (for an average sulfur loading of 1.2 mg cm^{-2}, the actual current is about 0.227 mA), so on the Land CT2001A program-controlled test system, The current per cell is set to 0.227 mA. The test results are as follows (Fig. S13).

In this discussion, we assume that the capacity (C_{All}) of the Li-S battery comes from the active material sulfur (C_S) and the separator material Ti_3C_2@CoS_2/CNTs (C_H), therefore,

$$C_{All} = C_S + C_H \tag{S2}$$

The masses of sulfur and Ti_3C_2@CoS_2/CNTs in Li-S battery are m_s and m_H, respectively. The specific capacities they contribute are SC_s and SC_H, respectively. In a typical calculation without considering the capacity contribution of Ti_3C_2@CoS_2/CNTs, the specific capacity of sulfur (SC) as shown in Fig. 5a, d in our manuscript is expressed as (All specific capacity calculations used in the manuscript are based on this formula),

$$SC = \frac{C_{All}}{m_S} \tag{S3}$$

However, the actual specific capacity of sulfur (SC_S) can be obtained by deducting the influence of the separator material,

$$SC_S = \frac{C_S}{m_S} = \frac{C_{All} - C_H}{m_S} = SC - SC_H \cdot \frac{m_H}{m_S} \tag{S4}$$

where $SC_H \cdot \frac{m_H}{m_S}$ is the additional capacity contributed by Ti_3C_2@CoS_2/CNTs. m_H and m_s are known for a certain battery. SC and SC_H have been given (Figs. 5a and S13). Additional capacity contributions can be easily calculated and deducted.

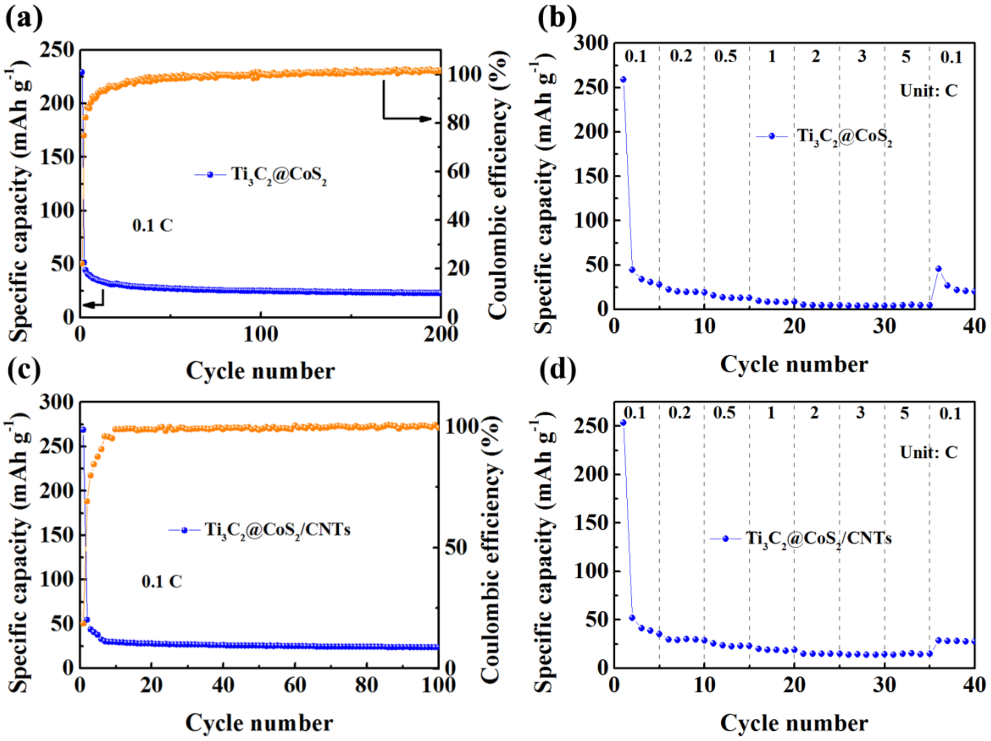

Fig. S13 a Cyclic performance, **b** rate performance of $Ti_3C_2@CoS_2$, **c** Cyclic performance, **d** rate performance of $Ti_3C_2@CoS_2$/CNTs

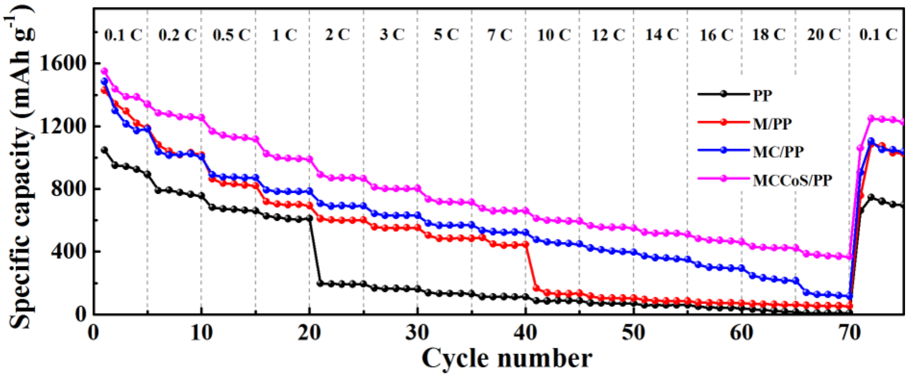

Fig. S14 Rate performance of the different separators after removing the contribution of separator capacity

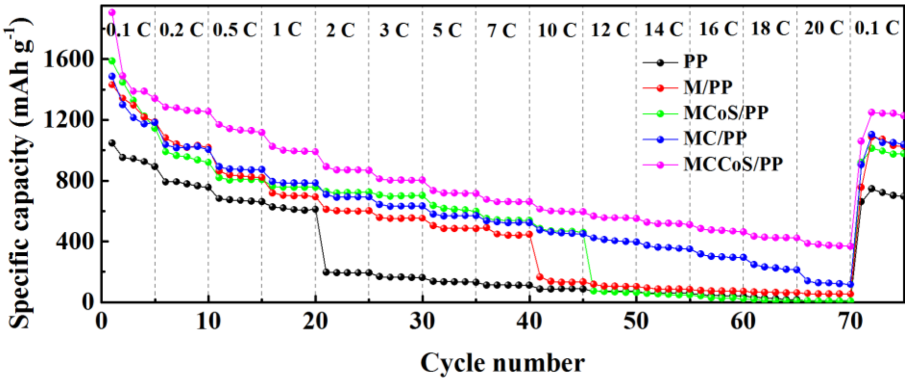

Fig. S15 rate performance of the different separators

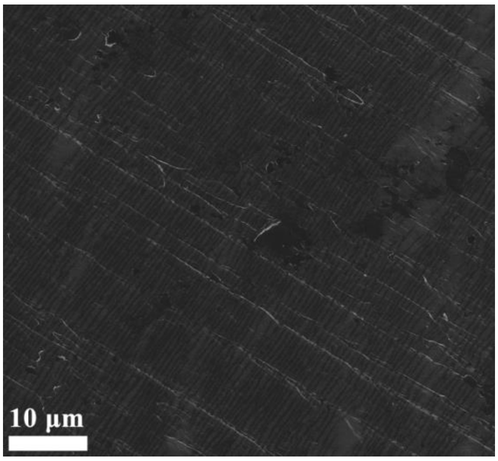

Fig. S16 SEM image of the surface of the unused lithium metal sheet

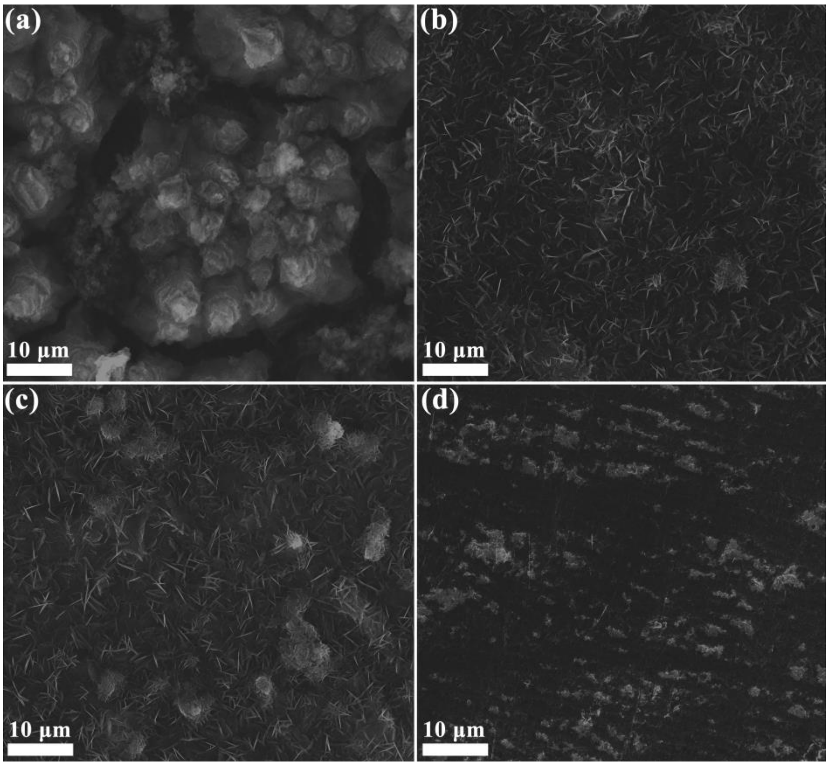

Fig. S17 SEM images of the surface of lithium sheets after high current cycling of Li-S batteries equipped with **a** PP, **b** M/PP, **c** MC/PP, **d** MCCoS/PP

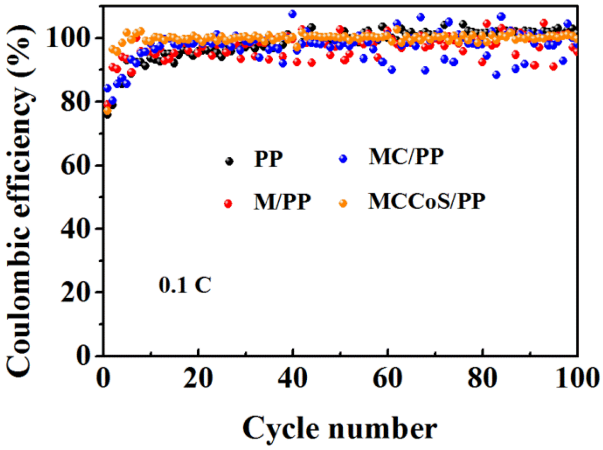

Fig. S18 Coulombic efficiencies of lithium-sulfur battery equipped with different separators

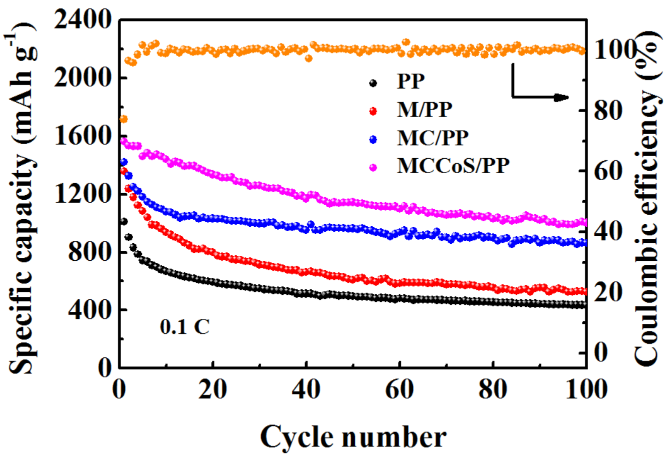

Fig. S19 Cyclic performance of the different separators after removing the contribution of separator capacity

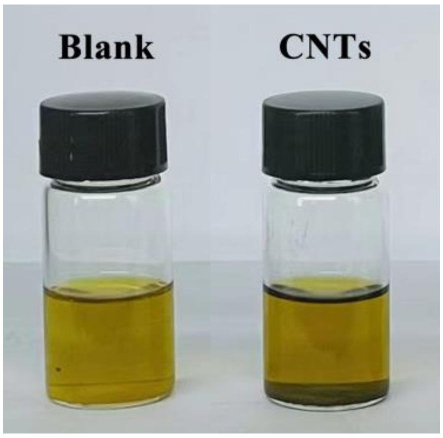

Fig. S20 Photo of CNTs soaked in a Li_2S_6 solution after 12h

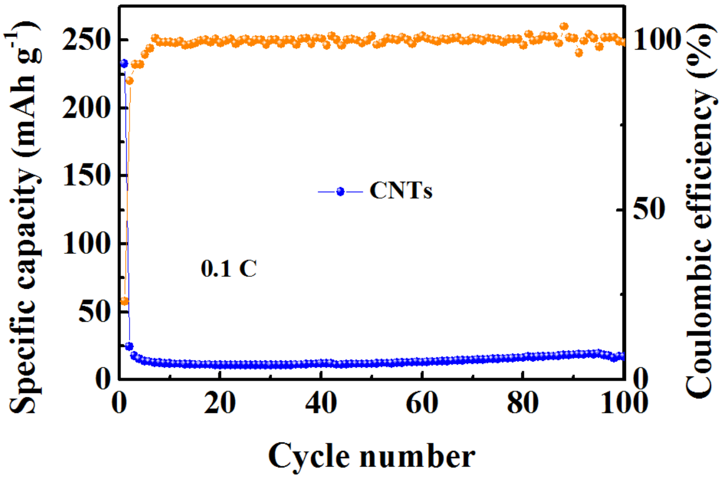

Fig. S21 Cyclic performance of CNTs as cathode material of Li-S battery

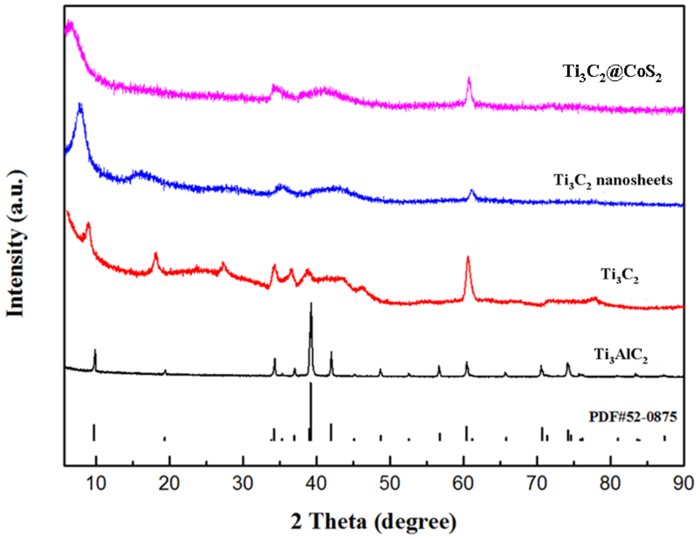

Fig. S22 XRD patterns of Ti_3AlC_2, Ti_3C_2, Ti_3C_2 nanosheets and Ti_3C_2@ CoS_2

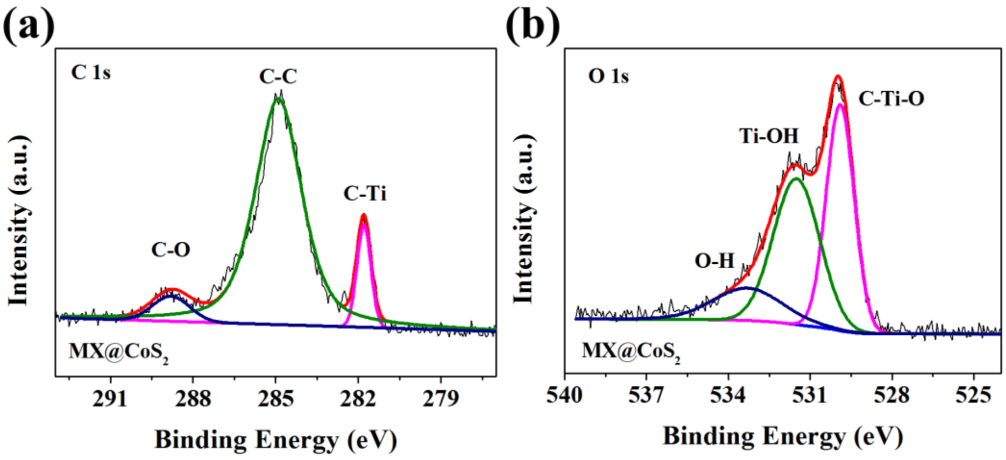

Fig. S23 a) XPS O 1s and **b**) XPS C 1s spectra of MX@CoS_2

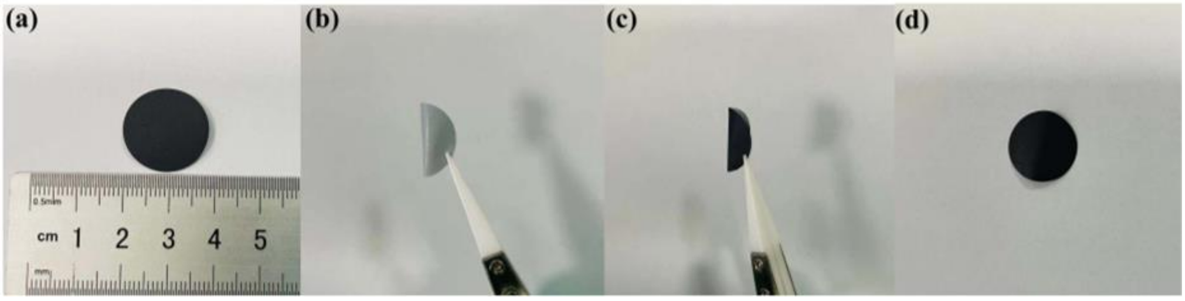

Fig. S24 a-d) Photographs of the MCCoS/PP modified separator

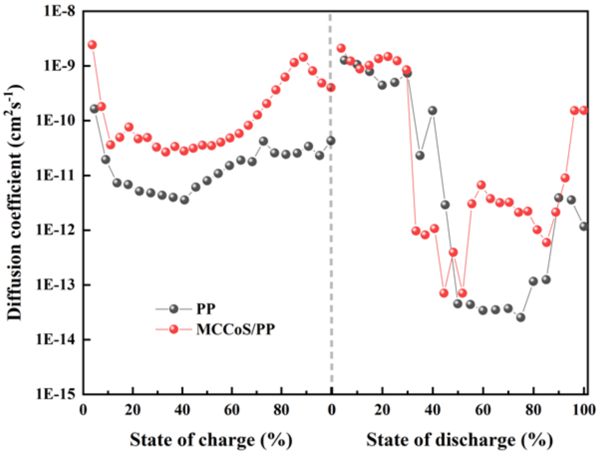

Fig. S25 GITT of of Li–S battery based on PP and MCCoS/PP separator

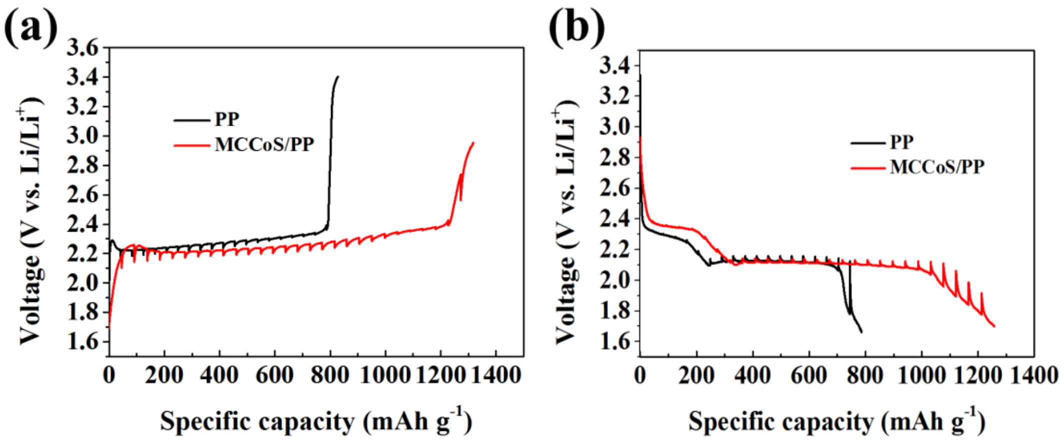

Fig. S26 a-b) GITT charge-discharge profiles of Li–S battery based on PP and MCCoS/PP separator

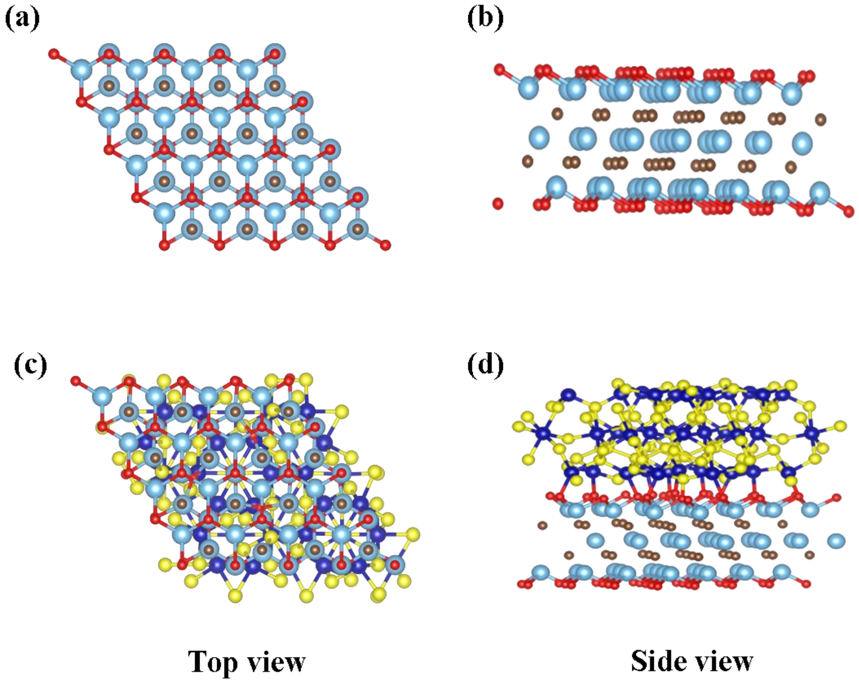

Fig. S27 Optimized structures of **a,b**) MX and **c,d**) MX@CoS_2

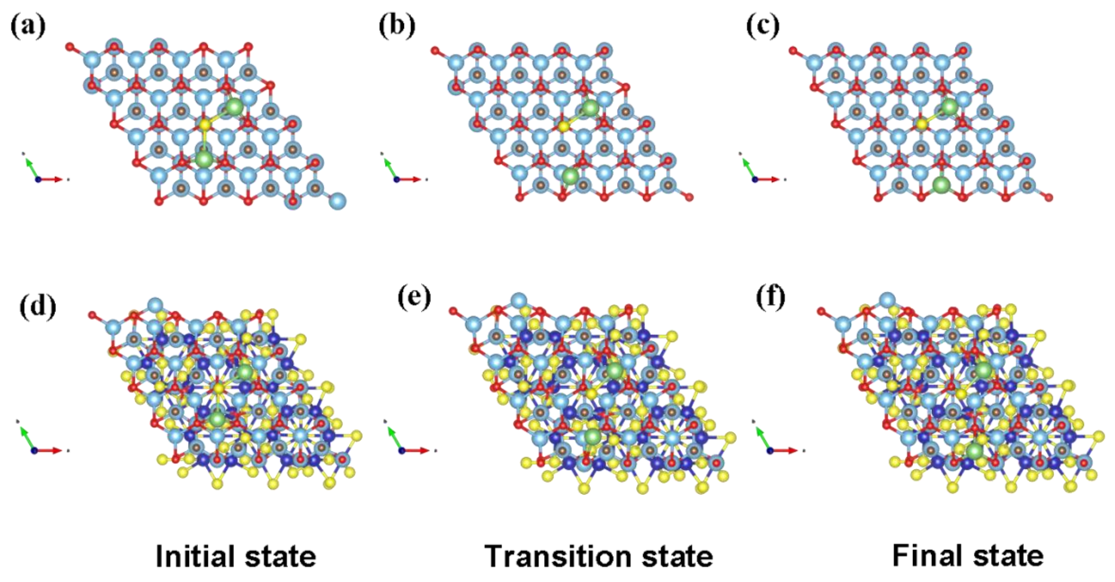

Fig. S28 Li_2S decomposition process of **a-c**) MX, **d-f**) MX@CoS_2

Table S1 Comparison of MCCoS/PP as separator of Li–S battery with state-of-the-art MX based materials

Host material	Capacity (mAh/g) (Low rate)	Capacity (mAh/g) (High rate)	Capacity retention (mAhg^{-1}/ %) (cycles, low rate)	Decay rate (per cycle,%) (time, rate)
MCCoS/PP<br>This work	1625.5<br>0.1C	368.6<br>20C	1002.3/61.7%<br>100 0.1C	0.033%<br>1000 7C
S@MXene@PDA<br>[S1]	1439<br>0.2C	624<br>6C	1044/73%<br>150 0.2C	0.048%<br>770 2C
Ti_3C_2/S and Ti_3C_2 interlayer<br>[S2]	1062<br>0.2C	288<br>10C	632/60%<br>50 0.5C	0.252%<br>200 2C
Crumpled N-doped $Ti_3C_2T_x$/S<br>[S3]	1144<br>0.2C	770<br>2C	950/83.1%<br>200 0.2C	
Porous N-doped $Ti_3C_2T_x$/S<br>[S4]	1072<br>0.5C	792<br>3C	1014/94.6%<br>100 0.5C	0.094%<br>600 5C
Mo_2C/CNTs/S<br>[S5]	1235<br>0.1C	665<br>5C	925/74.8%<br>250 0.1C	0.18%<br>100 5C
$Ti_3C_2T_x$ @ mesoporous C/S<br>[S6]	1225.8<br>0.05C	544.3<br>4C		
$Ti_3C_2T_x$ -1T-2H Mo_2S-C/S<br>[S7]	1194.7<br>0.1C	677.2<br>2C		0.07%<br>300 0.5C
CoS_2@NGCN/S<br>[S8]	1546<br>0.1C	525.3<br>2C	900/-<br>100 0.1C	0.075%<br>300 1C
CoS_2/HPGC/interlayer<br>[S9]	1055<br>0.2C	650<br>2C	846/80.1%<br>250 0.2C	0.07%<br>500 1C
TiN NM@C<br>[S10]		304<br>10C		0.059%<br>1000 4C
S/CoZn-Se@N-MX<br>[S11]	1270<br>0.2C	844<br>3C	1016/80%<br>100 0.2C	0.034%<br>2000 2C
PM-CNT- Separator	1105	677	801/72.5%	0.07%

[S12]	0.5C	2C	150 0.5C	500 1C
N-P $Ti_3C_2T_x$ /S<br>[S13]		819.5<br>2C		
PA-Mxene/CNT-50<br>[S14]		668<br>2C		0.025%<br>800 0.5C
OV-TnQDs@PCN/S<br>[S15]		674<br>2 C	878/90%<br>100 0.1C	0.012%<br>1000 2C
AB- CoS_2<br>[S16]	1108<br>0.2C	475<br>4 C	650/59%<br>150 0.2C	0.09%<br>450 2C
N- $Ti_3C_2T_x$/C@PP<br>[S17]	1363<br>0.1C	675<br>2C	1018/-<br>100 0.1C	0.07%<br>500 0.5C
$Ti_3C_2T_x$/GO@PP<br>[S18]		640<br>5C		0.23%<br>300 1C
MPF13-550/PP<br>[S19]	1235<br>0.1C	593<br>2C	721/61.7%<br>200 0.2C	0.375%<br>200 2C
CMP<br>[S20]	1415<br>0.1C	728<br>2C	992/70%<br>100 0.1C	0.06%<br>600 1C

Supplementary References

[S1] Y. Yao, W. Feng, M. Chen, X. Zhong, X. Wu et al., Boosting the electrochemical performance of Li–S batteries with a dual polysulfides confinement strategy. Small **14**(42), 1802516 (2018). https://doi.org/10.1002/smll.201802516

[S2] Y. Dong, S. Zheng, J. Qin, X. Zhao, H. Shi et al., All-MXene-based integrated electrode constructed by Ti_3C_2 nanoribbon framework host and nanosheet interlayer for high-energy-density Li–S batteries. ACS Nano **12**(3), 2381-2388 (2018). https://doi.org/10.1021/acsnano.7b07672

[S3] W. Bao, L. Liu, C. Wang, S. Choi, D. Wang et al., Facile synthesis of crumpled nitrogen-doped MXene nanosheets as a new sulfur host for lithium–sulfur batteries. Adv. Energy Mater. **8**(13), 1702485 (2018). https://doi.org/10.1002/aenm.201702485

[S4] Y. Song, Z. Sun, Z. Fan, W. Cai, Y. Shao et al., Rational design of porous nitrogen-doped Ti_3C_2 MXene as a multifunctional electrocatalyst for Li–S chemistry. Nano Energy **70**, 104555 (2020). https://doi.org/10.1016/j.nanoen.2020.104555

[S5] L. Lv, C. Guo, W. Sun, Y. Wang, Strong surface-bound sulfur in carbon nanotube bridged hierarchical Mo_2C-based MXene nanosheets for lithium–sulfur batteries. Small **15**(3), 1804338 (2019). https://doi.org/10.1002/smll.201804338

[S6] W. Bao, D. Su, W. Zhang, X. Guo, G. Wang, 3D metal carbide@mesoporous carbon hybrid architecture as a new polysulfide reservoir for lithium-sulfur batteries. Adv. Funct. Mater. **26**(47), 8746-8756 (2016). https://doi.org/10.1002/adfm.201603704

[S7] Y. Zhang, Z. Mu, C. Yang, Z. Xu, S. Zhang et al., Rational design of MXene/1T-2H MoS_2-C nanohybrids for high-performance lithium–sulfur batteries. Adv. Funct. Mater. **28**(38), 1707578 (2018). https://doi.org/10.1002/adfm.201707578

[S8] S. Seo, D. Park, S. Park, D. Kim, "Brain-coral-like" mesoporous hollow CoS_2@N-doped graphitic carbon nanoshells as efficient sulfur reservoirs for lithium–sulfur batteries. Adv. Funct. Mater. **29**(38), 1903712 (2019). https://doi.org/10.1002/adfm.201903712

[S9] Q. Hu, J. Lu, C. Yang, C. Zhang, J. Hu et al., Promoting reversible redox kinetics by separator architectures based on CoS_2/HPGC interlayer as efficient polysulfide-trapping shield for Li–S batteries. Small **16**(34), 2002046 (2020). https://doi.org/10.1002/smll.202002046

[S10] X. Huang, J. Tang, T. Qiu, R. Knibbe, Y. Hu et al., nanoconfined topochemical conversion from MXene to ultrathin non-layered tin nanomesh toward superior electrocatalysts for lithium-sulfur batteries. Small **17**(32), 2101360 (2021). https://doi.org/10.1002/smll.202101360

[S11] Z. Ye, Y. Jiang, L. Li, F. Wu, R. Chen, Self-assembly of 0D–2D heterostructure electrocatalyst from MOF and MXene for boosted lithium polysulfide conversion reaction. Adv. Mater. **33**(33), 2101204 (2021). https://doi.org/10.1002/adma.202101204

[S12] D. Xiong, S. Huang, D. Fang, D. Yan, G. Li et al., Porosity engineering of MXene membrane towards polysulfide inhibition and fast lithium ion transportation for lithium–sulfur batteries. Small **17**(34), 2007442 (2021). https://doi.org/10.1002/smll.202007442

[S13] C. Wei, M. Tian, Z. Fan, L. Yu, Y. Song et al., Concurrent realization of dendrite-free anode and high-loading cathode via 3D printed N-Ti_3C_2 MXene framework toward advanced Li–S full batteries. Energy Storage Mater. **41**, 141-151 (2021). https://doi.org/10.1016/j.ensm.2021.05.030

[S14] B. Zhang, C. Luo, G. Zhou, Z. Pan, J. Ma et al., Lamellar MXene composite aerogels with sandwiched carbon nanotubes enable stable lithium–sulfur batteries with a high sulfur loading. Adv. Funct. Mater. **31**(26), 2100793 (2021). https://doi.org/10.1002/adfm.202100793

[S15] H. Zhang, L. Yang, P. Zhang, C. Lu, D. Sha et al., MXene-derived Ti_nO_{2n-1} quantum dots distributed on porous carbon nanosheets for stable and long-life Li–S batteries: enhanced polysulfide mediation via defect engineering. Adv. Mater. **33**(21), 2008447 (2021). https://doi.org/10.1002/adma.202008447

[S16] P. Zeng, L. Huang, X. Zhang, Y. Han, Y. Chen, Inhibiting polysulfides diffusion of lithium-sulfur batteries using an acetylene black-CoS_2 modified separator: mechanism research and performance improvement. Appl. Surf. Sci. **427**, 242-252 (2018). https://doi.org/10.1016/j.apsusc.2017.08.062

[S17] G. Jiang, N. Zheng, X. Chen, G. Ding, Y. Li et al., In-situ decoration of MOF-derived carbon on nitrogen-doped ultrathin MXene nanosheets to multifunctionalize separators for stable Li-S batteries. Chem. Eng. J. **373**, 1309-1318 (2019). https://doi.org/10.1016/j.cej.2019.05.119

[S18] P. Liu, L. Qu, X. Tian, Y. Yi, J. Xia et al., $Ti_3C_2T_x$/graphene oxide free-standing membranes as modified separators for lithium–sulfur batteries with enhanced rate performance. ACS Appl. Energy Mater. **3**(3), 2708-2718 (2020). https://doi.org/10.1021/acsaem.9b02385

[S19] L. Lin, M. Qi, Z. Bai, S. Yan, Z. Sui et al., Crumpled nitrogen-doped aerogels derived from MXene and pyrrole-formaldehyde as modified separators for stable lithium-sulfur batteries. Appl. Surf. Sci. **555**, 149717 (2021). https://doi.org/10.1016/j.apsusc.2021.149717

[S20] N. Li, W. Cao, Y. Liu, H. Ye, K. Han, Impeding polysulfide shuttling with a three-dimensional conductive carbon nanotubes/MXene framework modified separator for highly efficient lithium-sulfur batteries. Colloids Surf. A Physicochem. Eng. Asp. **573**, 128-136 (2019). https://doi.org/10.1016/j.colsurfa.2019.04.054
